# Physiological and Transcriptional Analyses Provide Insight into Maintaining Ion Homeostasis of Sweet Sorghum under Salt Stress

**DOI:** 10.3390/ijms241311045

**Published:** 2023-07-03

**Authors:** Huan Guo, Chun-Ya Nie, Zhen Li, Jie Kang, Xiao-Long Wang, Yan-Nong Cui

**Affiliations:** College of Grassland Agriculture, Northwest A&F University, Yangling 712100, China; huan.guo@nwafu.edu.cn (H.G.); 17319697319@163.com (C.-Y.N.); lizhen135712@163.com (Z.L.); 15379989062@163.com (J.K.);

**Keywords:** soil salinity, sodium, chloride, ion transporters, transcription factors

## Abstract

Sweet sorghum is an important bioenergy grass and valuable forage with a strong adaptability to saline environments. However, little is known about the mechanisms of sweet sorghum coping with ion toxicity under salt stresses. Here, we first evaluated the salt tolerance of a sweet sorghum cultivar “Lvjuren” and determined its ion accumulation traits under NaCl treatments; then, we explored key genes involved in Na^+^, Cl^−^, K^+^ and NO_3_^−^ transport using transcriptome profiling and the qRT-PCR method. The results showed that growth and photosynthesis of sweet sorghum were unaffected by 50 and 100 mM NaCl treatments, indicative of a strong salt tolerance of this species. Under NaCl treatments, sweet sorghum could efficiently exclude Na^+^ from shoots and accumulate Cl^−^ in leaf sheaths to avoid their overaccumulation in leaf blades; meanwhile, it possessed a prominent ability to sustain NO_3_^−^ homeostasis in leaf blades. Transcriptome profiling identified several differentially expressed genes associated with Na^+^, Cl^−^, K^+^ and NO_3_^−^ transport in roots, leaf sheaths and leaf blades after 200 mM NaCl treatment for 6 and 48 h. Moreover, transcriptome data and qRT-PCR results indicated that *HKT1;5*, *CLCc* and *NPF7.3-1* should be key genes involved in Na^+^ retention in roots, Cl^−^ accumulation in leaf sheaths and maintenance of NO_3_^−^ homeostasis in leaf blades, respectively. Many *TFs* were also identified after NaCl treatment, which should play important regulatory roles in salt tolerance of sweet sorghum. In addition, GO analysis identified candidate genes involved in maintaining membrane stability and photosynthetic capacity under salt stresses. This work lays a preliminary foundation for clarifying the molecular basis underlying the adaptation of sweet sorghum to adverse environments.

## 1. Introduction

Soil salinity is one of the major environmental constraints on plant growth and crop production [1]. There are exceeding 1 billion ha lands affected by salinity worldwide [2]. In China, approximately 30 percent of the total 3.6 × 10^7^ ha salt-affected lands are potentially arable [3]. Therefore, with population expansion, urban spread and climate change, the use of salinized lands to cultivate crops and forages is an important strategy to ensure food security and promote ecological restoration [4].

Sweet sorghum [*Sorghum bicolor* (L.) Moench], an annual C_4_ plant belonging to Poaceae, is a natural variation of grain sorghum [5]. This species is characterized by high fermentable sugars in the juice of the stalks, which makes it attractive as a valuable bioenergy crop [6,7]. Meanwhile, sweet sorghum has been widely used as a forage due to its high biomass and growth rate, as well as prominent palatability and digestibility [8]. Furthermore, different from traditional crop species, sweet sorghum can adapt well to various environmental stresses including salinity, drought and flood, serving as a pioneer plant for recovering saline and marginal lands [5,9]. Combining these eminent traits, there have been increasing practices to evaluate field performance of sweet sorghum in salt-affected lands in China [2,3]. Thus, understanding of mechanisms employed by sweet sorghum to adapt to adverse environments will lay a theoretical basis for the large-scale cultivation of this species in salinized areas.

Salinity affects plant growth by directly imposing osmotic stress and ion toxicity or resulting in secondary stress such as oxidative stress and eventually restricting many biological processes such as photosynthesis, water uptake and nutrient acquisition or damaging the ultrastructure of plant cells [1,10]. Although researchers have analyzed growth and photosynthetic performance of sweet sorghum under salt stress and investigated the response of genes involved in photosynthesis, sugar biosynthesis and biological processes to salt treatments [11,12], the molecular mechanisms of sweet sorghum coping with ion toxicity are not well documented. Na^+^ and Cl^−^, the dominant inorganic ions in saline soil, are metabolically toxic to plants when they accumulate at high concentrations in the cytoplasm; therefore, they are closely associated with reductions in crop yield [13,14]. Plants mainly decrease toxic effects of Na^+^ and Cl^−^ by excluding or translocating them through ion transporters and channels [15,16]. It has been found that most species in Poaceae alleviate Na^+^-toxicity by maintaining a low Na^+^ content in leaves (termed Na^+^ exclusion trait) [4], which is predominately achieved by restricting the long-distance transport of Na^+^ from roots into shoots, and several key proteins involved in this process, such as HKT1;5 and HAK4, have been identified [17,18]. Physiological studies on wheat (*Triticum aestivum*) and rice (*Oryza sativa*) find that Cl^−^ content in leaves or shoots is much higher than that in roots under NaCl treatments [19,20], indicating that these species cannot efficiently restrict the transport of Cl^−^ into shoots under salt stress. Although the Cl^−^ tolerance in the model plant *Arabidopsis* and some Cl^−^-sensitive plants such as *Glycine max* and *Citus* spp. has been investigated and key proteins mediating Cl^−^ transport such as CLCs, NPFs and CCC1 in these plant species have been functionally characterized in recent years [21], the mechanisms underlying how Poaceae plants adapt to Cl^−^ toxicity still remain elusive.

The leaf sheath at the base of leaf tissue is a special structure evolved by Poaceae plants, which plays an essential role in supporting leaf blades, reserving nutrients and resisting chilling stress [22]. Furthermore, it has been proven that durum wheat (*Triticum turgidum* L. subsp. *Durum* Desf.) can accumulate large amounts of Na^+^ in leaf sheaths to decrease Na^+^ content in leaf blades under salt stresses [23], suggesting that the leaf sheath might act as a useful “Na^+^ reservoir” in shoots to avoid Na^+^ overaccumulation in leaf blades. However, the function of leaf sheaths in accumulation of other inorganic ions and alleviation of Cl^−^ toxicity to leaf blades has not been reported. Therefore, further study on the role of the leaf sheath in decreasing ion toxicity would provide new insights for understanding salt tolerance mechanisms of Poaceae plants.

Given the strong resistance of sweet sorghum to abiotic stresses, it is considered an important resource for exploring mechanisms and gene resources that can be used in the improvement of crop responses to environmental stresses [2]. Researchers have investigated ion accumulation characteristics in roots and shoots of sweet sorghum under saline conditions [24]. However, Na^+^ and Cl^−^ accumulation and distribution in leaf sheaths and leaf blades of this species have not been well reported. In addition, although transcription factors such as WRKY50 have been proven to play a key role in regulating Na^+^ transport in sweet sorghum under NaCl stress [25], the study of the function of Na^+^ and Cl^−^ transporters or channels and other transcription factors in salt tolerance of sweet sorghum still lags behind.

In this study, we first evaluated the physiological response of a sweet sorghum cultivar to NaCl treatments by measuring growth and photosynthesis indexes, then determined Na^+^, K^+^, Cl^−^ and NO_3_^−^ contents in roots, leaf sheaths and leaf blades under NaCl treatments. Subsequently, we investigated the expression changes of genes involved in ion transport and cellular components or genes encoding transcription factors after NaCl treatment by transcriptome sequencing. Finally, we analyzed expression patterns of *HKT1;5*, *CLCc* and *NPF7.3* in sweet sorghum after NaCl treatment using the qRT-PCR method.

## 2. Results

### 2.1. The Effect of NaCl Treatments on Growth and Photosynthesis of Sweet Sorghum

**Figure 1:**
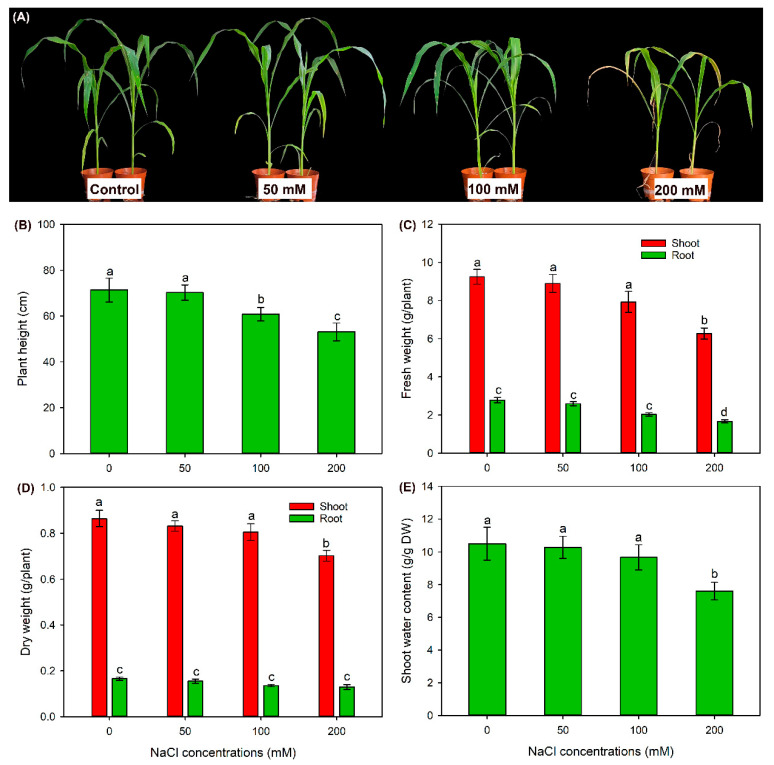
Effects of 50–200 mM NaCl treatments on growth of sweet sorghum cultivar “Lvjuren”. (**A**) Growth photograph, (**B**) plant height, (**C**) fresh weight, (**D**) dry weight and (**E**) shoot water content. Data are means (±SD), *n* = 6. Different letters indicate significant differences as determined using Tukey’s HSD test (*p* < 0.05).

After 50 and 100 mM NaCl treatments, leaf blades of seedlings were healthy, while leaf blades of 200 mM NaCl-treated seedlings were visually wilting (Figure 1A). Compared with the control, the 50 mM NaCl treatment had no effect on plant height (PH), fresh weight (FW) and dry weight (DW) of roots and shoots, as well as shoot water content (WC) (Figure 1B–E). The 100 mM NaCl treatment significantly decreased PH, while it had no effect on tissue biomass and shoot WC when compared with the control (Figure 1B–E). Differently, in comparison with the control, the 200 mM NaCl treatment sharply declined above-mentioned parameters (Figure 1B–E) (except for root DW, Figure 1D).

**Figure 2:**
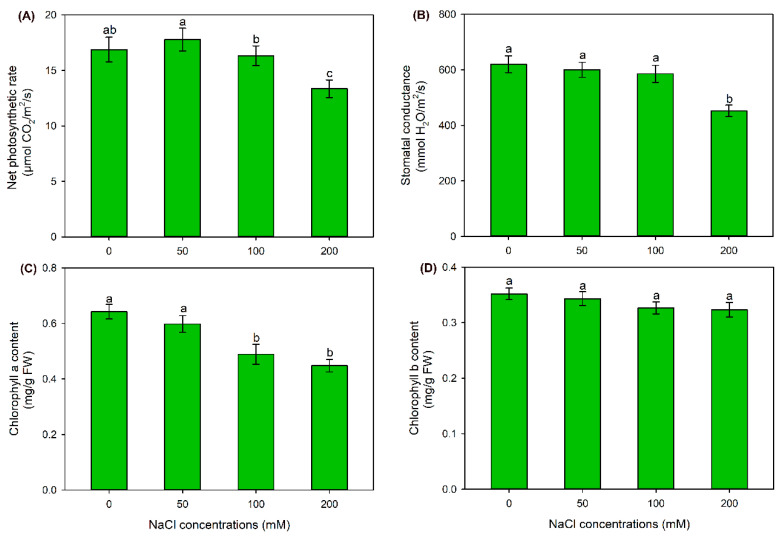
Effects of 50–200 mM NaCl treatments on photosynthesis of sweet sorghum cultivar “Lvjuren”. (**A**) Net photosynthetic rate, (**B**) stomatal conductance, (**C**) chlorophyll a content and (**D**) chlorophyll b content. Data are means (±SD), *n* = 6. Different letters indicate significant differences as determined using Tukey’s HSD test (*p* < 0.05).

The net photosynthetic rate (Pn) and stomatal conductance (Gs) under 50 and 100 mM NaCl treatments were maintained at the same level as those under the control condition; in contrast, Pn and Gs under 200 mM NaCl treatment were significantly decreased (Figure 2A,B). In comparison with the control, all the NaCl treatments had no effect on chlorophyll b content, but 100 and 200 mM NaCl treatments significantly decreased chlorophyll a content (Figure 2C,D). These results suggested that sweet sorghum cultivar “Lvjuren” could well tolerate 50 and 100 mM NaCl treatments, while its growth and photosynthesis were inhibited by the 200 mM NaCl treatment.

### 2.2. The Ion Contents in Different Tissues of Sweet Sorghum under NaCl Treatments

**Figure 3:**
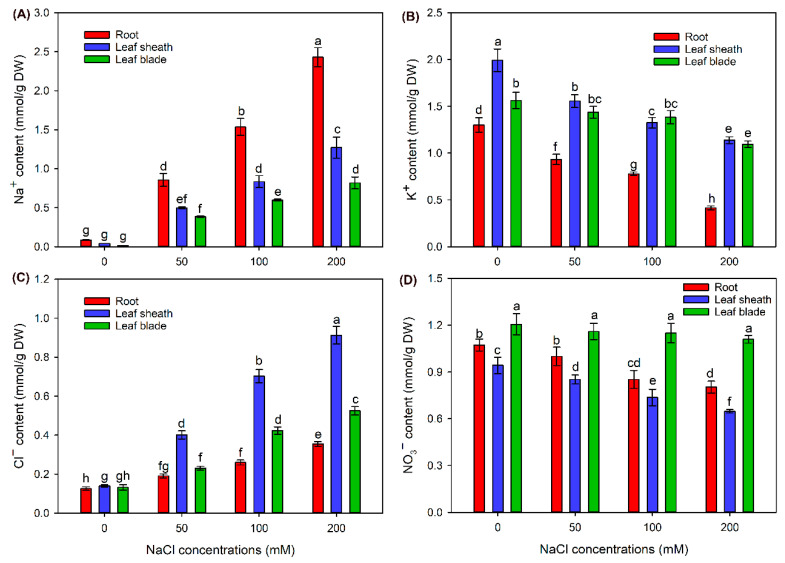
Effects of 50–200 mM NaCl treatments on tissue Na^+^ (**A**), K^+^ (**B**), Cl^−^ (**C**) and NO_3_^−^ (**D**) contents of sweet sorghum cultivar “Lvjuren”. Data are means (±SD), *n* = 6. Different letters indicate significant differences as determined using Tukey’s HSD test (*p* < 0.05).

Compared with the control, Na^+^ content in roots, leaf sheaths and leaf blades was gradually increased after 50–200 mM NaCl treatments (Figure 3A). It was obvious that Na^+^ content in roots was much higher than in leaf sheaths and leaf blades under NaCl treatments (Figure 3A). Meanwhile, in shoots, leaf sheath Na^+^ content under all treatments was significantly higher than leaf blade Na^+^ content (Figure 3A). In comparison with the control, all NaCl treatments significantly decreased K^+^ content in roots and leaf sheaths; differently, only the 200 mM NaCl treatment obviously decreased K^+^ content in leaf blades (Figure 3B).

After treatment with 50–200 mM NaCl, Cl^−^ content in all tissues was dramatically increased when compared with that under the control condition (Figure 3C). In contrast to the tissue Na^+^ distribution pattern, Cl^−^ content in roots was clearly lower than in leaf sheaths and leaf blades under 100 and 200 mM treatments (Figure 3C). Meanwhile, in shoots, Cl^−^ content in leaf sheaths was approximately two times higher than in leaf blades under all salt treatments (Figure 3C). Compared with the control, 100 and 200 mM NaCl treatments significantly decreased NO_3_^−^ content in roots and leaf sheaths, while all salt treatments had no effect on NO_3_^−^ content in leaf blades (Figure 3D).

### 2.3. RNA-Seq Analysis of Sweet Sorghum under NaCl Stress

After irrigation with Hoagland solution (C), or treatment with 200 mM NaCl (S) for 6 and 48 h, we collected root (R), leaf sheath (LS) and leaf blade (LB) samples for transcriptome sequencing. In total, 36 mRNA sequencing libraries (C6R1-3, S6R1-3, C6LS1-3, S6LS1-3, C6LB1-3, S6LB1-3, C24R1-3, S24R1-3, C24LS1-3, S24LS1-3, C24LB1-3, S24LB1-3) were finally generated. As shown in Table S2, in each library, at least 200 million clean reads were obtained by RNA-seq, with clean bases > 6.01 Gb and guanine-cytosine (GC) content > 51.5%. The quality score Q30 value of these libraries was more than 87.87% (Table S2). By mapping the clean reads in each library to the sorghum reference genome sequence (NCBI accession number: GCF_000003195.3), it was found that the percentage of mapped reads in these libraries was from 82.37% to 92.53% (Table S3). There were, in total, 5952 new genes (termed Sorghum_bicolor_newGene_1, 2, 3……) that could not be mapped to the reference genome sequence, among which 2574 members were functionally annotated by alignment against protein databases (Table S4).

### 2.4. Identification of DEGs Related to Ion Transport in Roots, Leaf Sheaths and Leaf Blades after NaCl Treatment for 6 and 24 h

After 200 mM NaCl treatment for 6 h, in total, 3843 (2355 upregulated, 1488 downregulated), 1895 (688 upregulated, 1207 downregulated) and 2355 (1409 upregulated, 946 downregulated) DEGs were identified in roots, leaf sheaths and leaf blades, respectively; after NaCl treatment for 48 h, in total, 1933 (1219 upregulated, 714 downregulated), 1270 (713 upregulated, 557 downregulated) and 4103 (1960 upregulated, 2143 downregulated) DEGs were identified in roots, leaf sheaths and leaf blades, respectively (Figure S1).

**Figure 4:**
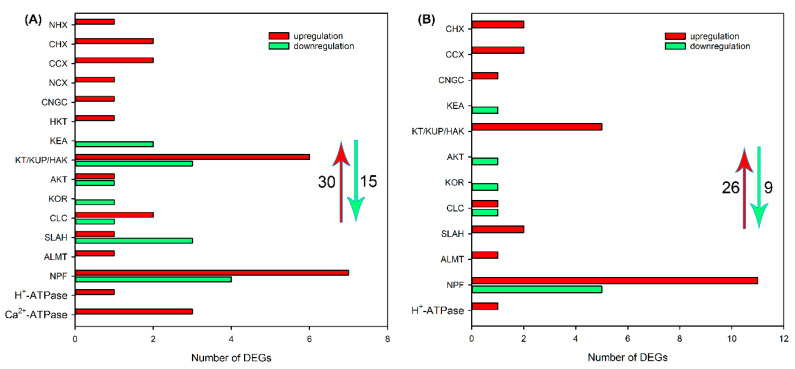
The number of DEGs related to Na^+^, K^+^, Cl^−^ and NO_3_^−^ transport in roots of sweet sorghum cultivar “Lvjuren” after 200 mM NaCl treatment for 6 (**A**) and 48 (**B**) h. Y axis indicates the upregulated and downregulated DEGs encoding ion transporters/channels. The red upward arrow and green downward arrow show the total number of upregulated DEGs and downregulated DEGs, respectively.

**Figure 5:**
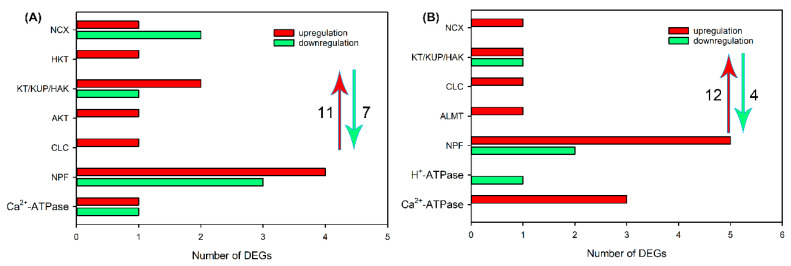
The number of DEGs related to Na^+^, K^+^, Cl^−^ and NO_3_^−^ transport in leaf sheaths of sweet sorghum cultivar “Lvjuren” after 200 mM NaCl treatment for 6 (**A**) and 48 (**B**) h, respectively. Y axis indicates the upregulated and downregulated DEGs encoding ion transporters/channels. The red upward arrow and green downward arrow show the total number of upregulated DEGs and downregulated DEGs, respectively.

**Figure 6:**
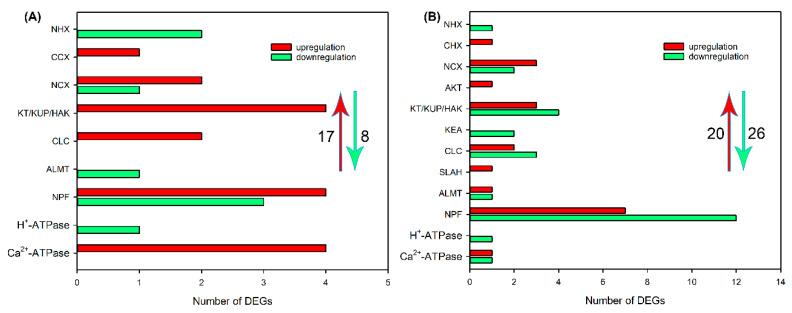
The number of DEGs related to Na^+^, K^+^, Cl^−^ and NO_3_^−^ transport in leaf blades of sweet sorghum cultivar “Lvjuren” after 200 mM NaCl treatment for 6 (**A**) and 48 (**B**) h. Y axis indicates the upregulated and downregulated DEGs encoding ion transporters/channels. The red upward arrow and green downward arrow show the total number of upregulated DEGs and downregulated DEGs, respectively.

Subsequently, we analyzed the effects of NaCl treatment on transcript levels of DEGs related to Na^+^, K^+^, Cl^−^ and NO_3_^−^ transport in different tissues. As shown in Figure 4A and Table S5, after NaCl treatment for 6 h, 30 upregulated DEGs, including *NHX*, *CHX*, *CCX*, *NCX*, *CNGC*, *HKT*, *KEA*, *HAK/KT/KUP*, *AKT* and *KOR* that are probably involved in Na^+^ and/or K^+^ transport, *CLC*, *SLAH*, *ALMT*, *NPF* that are probably involved in Cl^−^ and/or NO_3_^−^ transport and *H^+^-ATPase* and *Ca*^2+^*-ATPase* that provide H^+^ or Ca^2+^ pumps for ion transport, and 15 downregulated DEGs, including *KEA*, *HAK/KT/KUP*, *AKT*, *KOR*, *CLC*, *SLAH* and *NPF*, were identified in roots of sweet sorghum. After NaCl treatment for 48 h, the number of DEGs related to ion transport in roots declined, but 1 *CNGC*, *CLC*, *ALMT* and *H^+^-ATPase*; 2 *CHX*, *CCX* and *SLAH*; 5 *HAK/KT/KUP;* and 11 *NPF* were still upregulated (Figure 4B and Table S6). In leaf sheaths, after NaCl treatment for 6 and 48 h, only 11 and 12 upregulated DEGs related to Na^+^, K^+^, Cl^−^ and NO_3_^−^ transport were identified, respectively, and several members that were detected in roots, such as *NHX*, *CHX*, *CNGC*, *KEA*, *KOR*, *SLAH* and *ALMT,* were not identified (Figure 5, Tables S7 and S8). In leaf blades, after NaCl treatment for 6 and 48 h, 17 and 20 upregulated DEGs, as well as 8 and 26 downregulated DEGs, respectively, were identified (Figure 6, Tables S9 and S10). It was found that some DEGs such as *AKT*, *KEA* and *SLAH* were only detected in leaf blades under NaCl treatment for 48 h (Figure 6, Tables S9 and S10), suggesting that these genes might be mainly responsible for long-term salt stress.

**Figure 7:**
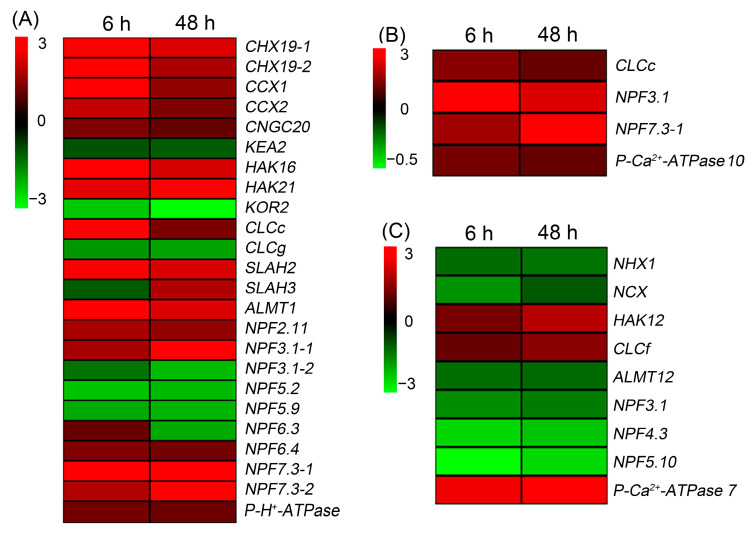
Heat maps showing the expression changes of DEGs related to ion transport that were detected in roots (**A**), leaf sheaths (**B**) and leaf blades (**C**) after 200 mM NaCl treatment for both 6 and 48 h.

Finally, we further screened the DEGs related to ion transport that can be detected under NaCl treatment for both 6 and 48 h. The heat map showed that, in roots, there were 24 DEGs related to ion transport after NaCl treatment for both 6 and 48 h, and the majority of these DEGs were upregulated (Figure 7A). In leaf sheaths, only four upregulated DEGs (*CLCc*, *NPF3.1*, *NPF7.3-1* and *P*-*Ca*^2+^*-ATPase 10*) were identified after NaCl treatment for both 6 and 48 h (Figure 7B). In leaf blades, although nine DEGs were identified after NaCl treatment for both 6 and 48 h, only three members (*HAK12*, *CLCf* and *P*-*Ca*^2+^*-ATPase 7*) were upregulated (Figure 7C).

### 2.5. Identification of DEGs Encoding Transcription Factors in Roots, Leaf Sheaths and Leaf Blades after NaCl Treatment for 6 h

**Figure 8:**
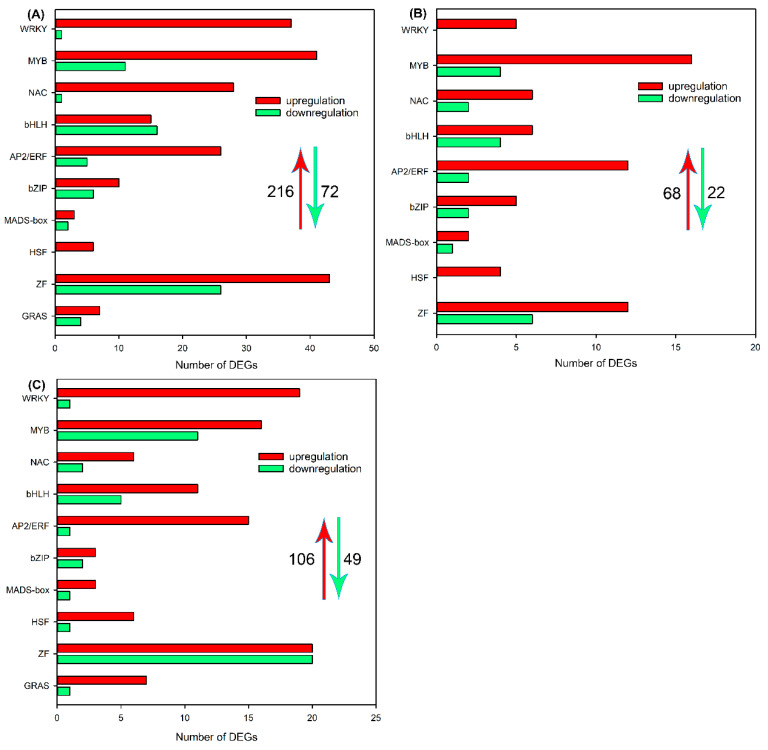
The number of DEGs encoding transcription factors in roots (**A**), leaf sheaths (**B**) and leaf blades (**C**) after 200 mM NaCl treatment for 6 h. Y axis indicates the upregulated and downregulated DEGs encoding transcription factors. The red upward arrow and green downward arrow show the total number of upregulated DEGs and downregulated DEGs, respectively.

As the expression of transcription factor genes (*TFs*) changes rapidly in response to abiotic stresses [26], we analyzed differentially expressed *TFs* in tissues of sweet sorghum after salt treatment for 6 h. As shown in Figure 8A, 216 upregulated *TFs* and 72 downregulated *TFs* were identified in roots, and these DEGs were categorized into *WRKY*, *MYB*, *NAC*, *bHLH*, *AP2/ERF*, *bZIP*, *MADS-box*, *HSF*, *ZF* and *GRAS* families. In leaf sheaths and leaf blades, the number of differentially expressed *TFs* was less than in roots (Figure 8B,C). Moreover, it was noticed that the numbers of upregulated *TFs* in all tissues were much more than downregulated *TFs* (Figure 8).

**Figure 9:**
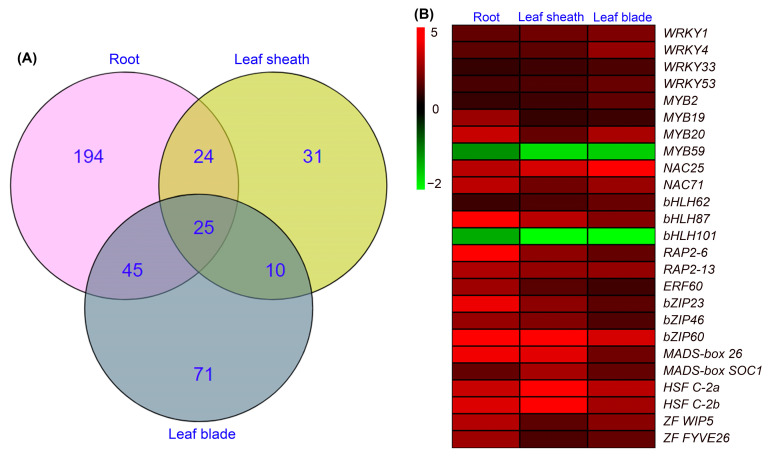
Venn diagram showing the number of exclusive and common DEGs encoding transcription factors in roots, leaf sheaths and leaf blades (**A**); heat map showing the expression changes of DEGs encoding transcription factors in all tissues after 200 mM NaCl treatment for 6 h (**B**).

The Venn diagram shows that, among the above differentially expressed *TFs*, 194, 31 and 71 members were specifically identified in roots, leaf sheaths and leaf blades, respectively (Figure 9A). As 25 differentially expressed *TFs* were detected in all 3 tissues (Figure 9A), we then analyzed the expression changes of these *TFs* and found that only 2 members (*MYB59* and *bHLH101*) in roots, leaf sheaths and leaf blades were downregulated, while the other 23 members in all tissues were upregulated (Figure 9B), which indicated that these *TFs* might play key roles in the salt tolerance of sweet sorghum. 

### 2.6. GO Analysis on DEGs Involved in Cellular Component

As shown in Figures S2–S7, after salt treatment for 6 or 48 h, several DEGs categorized into cellular component were identified in roots, leaf sheaths and leaf blades of sweet sorghum by GO analysis. It was found that, in roots for 6 h, the majority of GO termed DEGs were categorized into integral component of membrane, plasma membrane and membrane (Figure S2), and in roots for 48 h, intracellular membrane-bound organelle, cytosolic large ribosomal subunit and nucleolus were the most abundant GO terms (Figure S3). In leaf sheaths for both 6 and 48 h, the majority of GO-termed DEGs were also categorized into integral component of membrane and plasma membrane (Figures S4 and S5). In leaf blades for both 6 and 48 h, almost all the GO-termed DEGs were associated with photosynthesis, such as chloroplast, plastid and thylakoid (Figures S6 and S7). 

### 2.7. Validation of RNA-Seq Results

To verify RNA-seq data, the relative expression levels of 20 randomly selected genes were analyzed by the qRT-PCR method. Then, the correlation between RNA-seq results and qRT-PCR results was determined. As shown in Figure S8, the R^2^ in roots, leaf sheaths and leaf blades after salt treatment for 6 and 48 h was more than 0.89, indicating that the RNA-seq data were reliable.

### 2.8. Expression Pattern of HKT1;5, CLCc and NPF7.3-1 in Sweet Sorghum under NaCl Treatments

**Figure 10:**
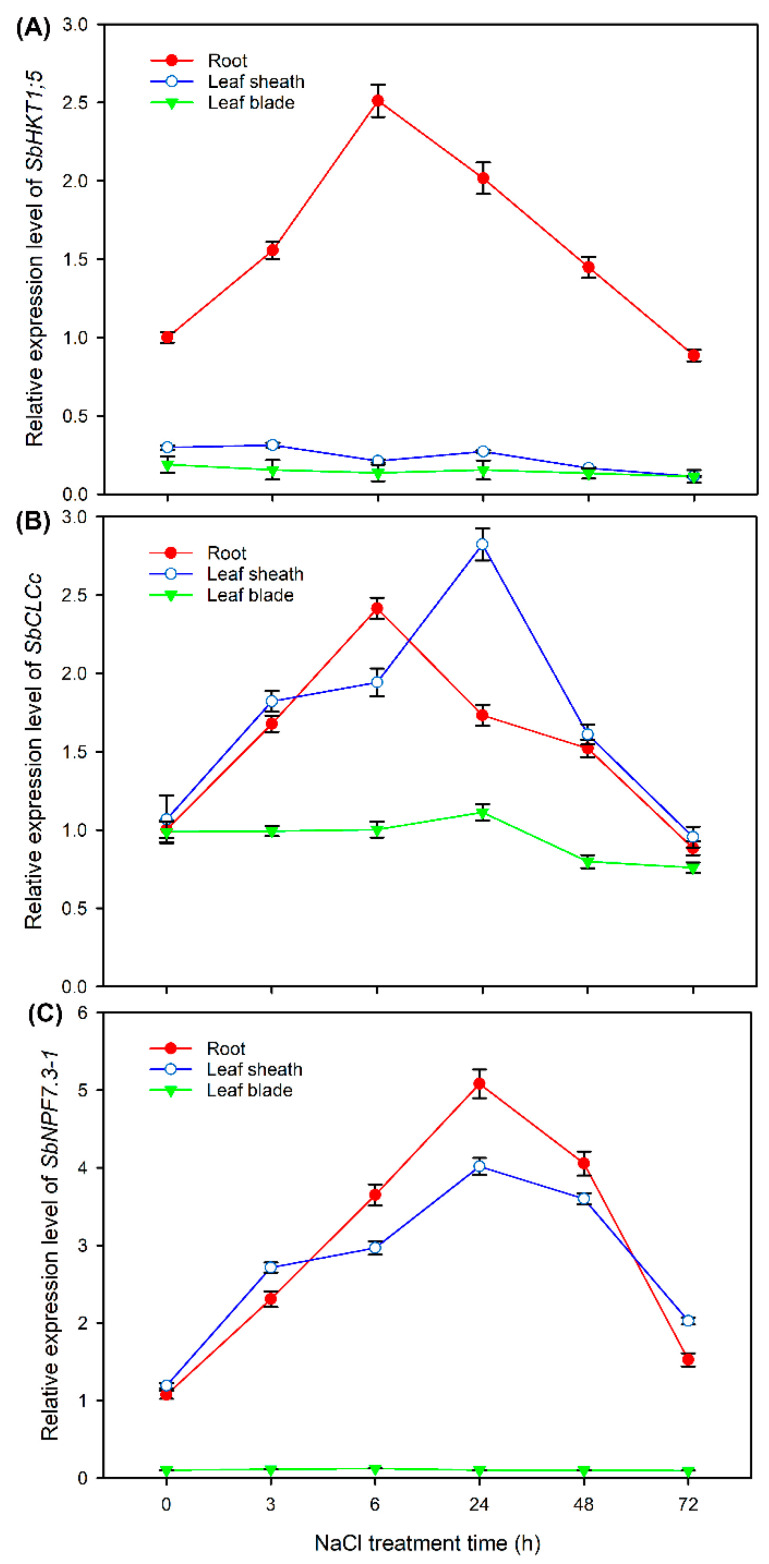
The relative expression levels of *SbHKT1;5* (**A**), *SbCLCc* (**B**) and *SbNPF7.3-1* (**C**) in roots, leaf sheaths and leaf blades of sweet sorghum cultivar “Lvjuren” after 200 mM NaCl treatment for 0, 3, 6, 24, 48 and 72 h. Data are means (±SD), *n* = 3.

As shown in Figure 10A, under normal conditions (NaCl treatment for 0 h), *SbHKT1;5* was mainly expressed in roots; moreover, its relative expression level in roots was increased after NaCl treatment for 3 and 6 h, then it gradually decreased with prolonged treatment time. Although *SbCLCc* showed no tissue-specific expression under normal conditions, its expression was induced only in roots and leaf sheaths by NaCl treatment for 3–48 h (Figure 10B). *SbNPF7.3-1* was dominantly expressed in roots and leaf sheaths; it was noticed that the expression of this gene in roots and leaf sheaths was sharply increased (more than 3-fold) after NaCl treatment for 6–48 h (Figure 10C).

## 3. Discussion

### 3.1. Sweet Sorghum Could Efficiently Exclude Na^+^ from Shoots and Accumulate Cl^−^ in Leaf Sheaths under NaCl Stress

The ability to maintain a low Na^+^ content in shoots or leaves, which is termed the Na^+^ exclusion trait, is vital for the salt tolerance of plant species in Poaceae [4,27]. In this study, Na^+^ content in shoots, especially in leaf blades, was much lower than that in roots of sweet sorghum under 50–200 mM NaCl treatments (Figure 3A). Differently, a study on grain sorghum cultivars has shown that Na^+^ content in leaf blades is close to or even higher than in roots under 200 mM NaCl treatment [28]. The Na^+^ exclusion trait is mainly achieved by the retrieval of Na^+^ from root xylem sap to restrict Na^+^ transport from roots into shoots [17,18]. Therefore, in comparison with grain sorghum, sweet sorghum possesses a stronger ability to restrict the long-distance transport of Na^+^ under salt stress. It has been reported that when Na^+^ is translocated into shoots of durum wheat, leaf sheaths could accumulate the majority of Na^+^ to decrease Na^+^ content in leaf blades [23]. Our results also showed that Na^+^ content in leaf sheaths was clearly higher than that in leaf blades of sweet sorghum under NaCl treatments (Figure 3A), suggesting that the retention of Na^+^ in leaf sheaths should be also important for sweet sorghum coping with Na^+^ toxicity to leaf blades. 

Studies have found that Cl^−^ content in shoots of wheat, rice and grain sorghum is higher than that in roots under NaCl stress [19,20,28], suggesting that Poaceae plants should not evolve the Cl^−^ exclusion trait from shoots. However, the mechanisms of plants in this family coping with Cl^−^ toxicity are still not clear so far. Wei et al. [24] found that sweet sorghum could also accumulate much more Cl^−^ in shoots than in roots under salt stress. Similarly, in this study, Cl^−^ content in leaf sheaths and leaf blades of sweet sorghum was clearly higher than that in roots under NaCl treatments (Figure 3C). These results suggest that sweet sorghum could transport the majority of Cl^−^ into aerial parts under saline conditions. As the equilibrium potential of cell membranes is negative [29], the accumulation of Cl^−^ in aerial parts might help to balance the positive charge of Na^+^ for the maintenance of membrane stability. It was observed that Cl^−^ content in leaf sheaths was nearly two-fold higher than that in leaf blades under NaCl treatments (Figure 3C), indicating that leaf sheaths of sweet sorghum also serve as an indispensable “Cl^−^ reservoir” to avoid Cl^−^ overaccumulation in leaf blades under salt stresses. Therefore, the large accumulation of Cl^−^ in leaf sheaths should be a key process of sweet sorghum alleviating Cl^−^ toxicity.

K^+^ and NO_3_^−^ are essential macronutrients for plant growth and both act as important inorganic osmotica [1,30]. Because Na^+^ and Cl^−^ would compete for biding sites of K^+^ and NO_3_^−^ transporters or channels, the uptake and accumulation of K^+^ and NO_3_^−^ in most glycophytes are severely inhibited under salt stress [31,32]. Differently, the K^+^ content in leaves of the halophyte *Atriplex canescens* is unaffected by 400 mM NaCl treatment, and the NO_3_^−^ content in shoots of the xerophyte *Pugionium cornutum* can also be maintained at high levels under salt stresses [30,33]. Therefore, the maintenance of K^+^ and NO_3_^−^ homeostasis is vital for the salt tolerance of plants. In this study, K^+^ content in leaf blades was maintained relatively stable under 50 and 100 mM NaCl treatments, while significantly declined under 200 mM NaCl (Figure 3B), suggesting that sweet sorghum could maintain K^+^ homeostasis in leaf blades under low and moderate salt stresses. Interestingly, NO_3_^−^ content in leaf blades under 50–200 mM NaCl treatments was the same as that under the control condition (Figure 3D), indicative of a prominent ability for maintaining NO_3_^−^ homeostasis in leaf blades of sweet sorghum under severe salt stresses.

### 3.2. The Genes Related to Ion Transport Play Key Roles in the Salt Tolerance of Sweet Sorghum

It has been found that some wild plant species such as halophytes and xerophytes evolve diverse adaptative mechanisms to cope with ion toxicity; for example, the halophyte *Sueada salsa* and xerophyte *Zygophyllum xanthoxylum* can accumulate high quantities of Na^+^ in the vacuoles of their succulent leaves to decrease the disturbance on cell metabolism and enhance cell osmotic adjustment ability [34,35]; the salt-secreting halophytes *Reaumuria trigyna* and *Chenopodium quinoa* can efficiently export Na^+^ and Cl^−^ via the salt glands and salt bladders on the leaf surface, respectively [36,37]. However, for most glycophytes that do not evolve succulent tissues or salt-secreting organs, the alleviation of Na^+^ and Cl^−^ toxicity on photosynthetic organs is mainly achieved by excluding them from roots or decreasing the long-distance transport of them from roots into shoots, and these processes are dominated by ion transporters and channels [15,21]. In this study, we identified many DEGs related to Na^+^ and Cl^−^ transport including *NHX*, *CHX*, *CCX*, *NCX*, *CNGC*, *HKT*, *CLC, SLAH*, *ALMT* and *NPF* in different tissues of sweet sorghum after 200 mM NaCl treatment by transcriptome sequencing (Figure 4, Figure 5 and Figure 6). These genes probably play key roles in sweet sorghum coping with ion toxicity. It was obvious that the number of DEGs in roots was more than that in leaf sheaths and leaf blades (Figure 4, Figure 5 and Figure 6), suggesting that the root primarily controls ion transport under salt stresses. HKT1;5 and HAK4 are thought to be involved in restricting long-distance transport of Na^+^ from roots into shoots in rice and maize by mediating the retrieval of Na^+^ from root xylem sap [17,18]. In our transcriptome data, no expression change of HAK4 encoding gene in sweet sorghum was found, however, the expression of HKT1;5 encoding gene in roots was upregulated after salt treatment for 6 h (Table S5). Moreover, qRT-PCR results showed that the relative expression level of *HKT1;5* in roots was substantially increased under NaCl treatment for 3–24 h (Figure 10A). Taken together, HKT1;5 should play a key role in the Na^+^ exclusion trait of sweet sorghum.

Our physiological results showed that the leaf sheath of sweet sorghum can accumulate large amounts of Na^+^ and Cl^−^ to restrict their transport into leaf blades (Figure 3). It has been reported that HKT1;4 functions in the retention of Na^+^ in leaf sheaths of durum wheat under saline conditions [38]. Interestingly, our transcriptome data showed that the transcript of *HKT1;4* was only detected in leaf sheaths of sweet sorghum; moreover, its expression was upregulated after NaCl treatment for 6 h (Table S7), suggesting that HKT1;4 should also play a key role in Na^+^ accumulation in leaf sheaths of sweet sorghum. The vacuolar sequestration of Cl^−^ mediated by chloride channel CLC dominates the accumulation of Cl^−^ in plant tissues [39,40]. In our transcriptome data, the expression of CLCc encoding gene in leaf sheaths of sweet sorghum was upregulated after NaCl treatment for both 6 and 48 h (Figure 7A). Meanwhile, the relative expression levels of *CLCc* in roots and leaf sheaths showed an increasing trend under NaCl treatment for 3–48 h (Figure 10B), indicating that CLCc should play a vital role in the accumulation of Cl^−^ in roots and leaf sheaths of sweet sorghum under saline conditions. Except for *HKT1;4* and *CLCc*, we also identified many DEGs related to Na^+^ and Cl^−^ transport in leaf sheaths, such as *NCX* and *NPF* (Figure 5), and these genes might be also involved in the Na^+^ and Cl^−^ accumulation in leaf sheaths of sweet sorghum.

The sequestration of Na^+^ and Cl^−^ in vacuoles of photosynthetic organs is essential for the salt tolerance of plants [41,42]. The tonoplast-located NHX (e.g., NHX1 and NHX2) are key proteins mediating the transport of Na^+^ into vacuoles [43,44]. However, in this study, the expression of NHX1 and NHX2 encoding genes in leaf blades of sweet sorghum was downregulated after NaCl treatment for 6 and 24 h (Tables S9 and S10), suggesting that there might be other molecular components involved in vacuolar sequestration of Na^+^ in leaf blades of sweet sorghum. In *Arabidopsis* and *P. cornutum*, CLCg is proven to mediate vacuolar Cl^−^ sequestration in shoots [40,42]. In our transcriptome data, two transcripts of *CLCg* (named *SbCLCg-1* and *SbCLCg-2*) were identified in sweet sorghum, and the expression of *SbCLCg-1* was upregulated, while the expression of *SbCLCg-2* was downregulated in leaf blades after NaCl treatment for 48 h (Table S10). Therefore, SbCLCg-1 should be an indispensable transporter mediating the sequestration of Cl^−^ in cell vacuoles of leaf blades in sweet sorghum under salt stresses.

Sweet sorghum possesses a prominent ability to maintain NO_3_^−^ homeostasis in leaf blades under salt stresses (Figure 3D). In the model plant *Arabidopsis*, NPF7.3 is thought to mediate NO_3_^−^ loading into root xylem and, therefore, is involved in the long-distance transport of NO_3_^−^ from roots into shoots [45]. However, the expression of *NPF7.3* in roots of *Arabidopsis* is suppressed by salt stress, which is the primarily reason why the NO_3_^−^ content in shoots of *Arabidopsis* is substantially declined under NaCl treatments [46]. Differently, in our transcriptome data, two transcripts of *NPF7.3* (named *NPF7.3-1* and *NPF7.3-2*) were identified, and the expressions of both genes in roots were upregulated after NaCl treatment for both 6 and 48 h (Figure 7A), suggesting that sweet sorghum could enhance the translocation of NO_3_^−^ into shoots by upregulating the expression of NPF7.3-1 and NPF7.3-2 encoding genes in roots. Moreover, the expression of NPF7.3-1 encoding gene in leaf sheaths was also upregulated after salt treatment for both 6 and 48 h (Figure 7B), and qRT-PCR results verified that its expression level in leaf sheaths sharply increased under NaCl treatment for 3–48 h (Figure 10C). Given that NPF7.3 mediates NO_3_^−^ efflux at the cellular level [45], we speculate that when NO_3_^−^ is transported into shoots of sweet sorghum, NPF7.3-1 functions in the efflux of NO_3_^−^ from leaf sheath cells, thus helping the transport of NO_3_^−^ into leaf blades. Although the K^+^ contents in roots, leaf sheaths and leaf blades of sweet sorghum were significantly decreased under 200 mM NaCl treatment (Figure 3B), transcriptome sequencing identified many DEGs related to K^+^ transport, such as *KEA*, *KT/KUP/HAK*, *AKT* and *KOR* (Figure 4, Figure 5 and Figure 6), suggesting that these genes should also play essential roles in maintaining K^+^ absorption and accumulation in sweet sorghum under saline conditions.

It has been reported that the expressions of *H^+^-ATPase* and *Ca*^2+^*-ATPase* in roots and shoots of *P. cornutum* were upregulated under salt stresses [26]. Similarly, in this study, the expressions of several *H^+^-ATPase* and *Ca*^2+^*-ATPase* were upregulated in roots, leaf sheaths and leaf blades of sweet sorghum after NaCl treatments (Figure 4, Figure 5 and Figure 6), suggesting that these ATPase should provide H^+^ and Ca^2+^ pumps for the transmembrane transport of ions such as Na^+^, K^+^, Cl^−^ and NO_3_^−^ and, therefore, are also involved in the maintenance of ion homeostasis of sweet sorghum under salt stresses.

### 3.3. Identification of Key Transcription Factors Involved in the Salt Tolerance of Sweet Sorghum

*TFs* are important regulatory genes involved in plant adaptations to environmental stresses [47]. Recent studies have reported the function of *TFs* in the salt tolerance of sweet sorghum, for example, *SbWRKY50* could drive the expression of *SOS1* and *HKT1* to regulate the ion homeostasis, and *SbbHLH85* enhances the Na^+^ absorption by roots [25,48]. To explore other candidate *TFs* regulating the response of sweet sorghum to soil salinity, in this study, we analyzed differentially expressed *TFs* after NaCl treatment for 6 h by transcriptome sequencing. Our results identified hundreds of *TFs* including *WRKY*, *MYB*, *NAC*, *bHLH*, *AP2/ERF*, *bZIP*, *MADS-box*, *HSF*, *ZF* and *GRAS* in roots, leaf sheaths and leaf blades of sweet sorghum, and the majority were upregulated after salt treatment (Figure 8). Furthermore, 25 differentially expressed *TFs* were detected in all tissues (Figure 9), and these *TFs* might play essential regulatory roles in the adaptation of sweet sorghum to salt stresses. Although a previous study has identified differentially expressed *TFs* in shoots of sweet sorghum after 200 mM NaCl treatment [12], the response of *TFs* in leaf sheaths of sweet sorghum to salt stress has not been well documented. In this study, we identified 68 upregulated *TFs* and 22 downregulated *TFs* in leaf sheaths of sweet sorghum after 200 mM NaCl treatment for 6 h (Figure 8B), which should play key roles in regulating the expression of function genes in leaf sheaths. Moreover, in the present study, Venn diagrams showed that 31 differentially expressed *TFs* were identified exclusively in leaf sheaths but not in roots or leaf blades after NaCl treatment (Figure 9A). Given that the leaf sheath plays an indispensable role in maintaining ion homeostasis of sweet sorghum, further study on the function of these *TFs* is likely to provide new insights into the salt tolerance of sweet sorghum. 

### 3.4. Sweet Sorghum Possesses a Strong Ability to Maintain Membrane Stability and Photosynthetic Ability under Salt Stresses

The maintenance of cellular organelle characteristics is essential for higher plants to adapt to environmental stresses [10]. For plants grown in saline conditions, osmotic stress and ion toxicity result in a large formation of reactive oxygen species (ROS), which could primarily damage cell membranes, organelles and nucleolus and, as a consequence, trigger oxidative stress on plant growth [49]. In the present study, we analyzed DEGs involved in cellular components in roots, leaf sheaths and leaf blades after 200 mM NaCl treatment using GO annotation. Our results showed that in roots and leaf sheaths, the number of DEGs categorized into integral component of membrane was clearly much higher than other GO terms after 200 mM NaCl treatment for 6 and 24 h (Figures S2–S5), suggesting that sweet sorghum could maintain membrane stability in roots and leaf sheaths by regulating the expression of these genes under salt stresses. It was noticed that the number of DEGs related to membrane terms in roots after NaCl treatment for 48 h was much lower than that after NaCl treatment for 6 h, and many members after 48 h treatment were categorized into ribosomal subunit and nucleolus (Figures S2 and S3), indicating that under long-term salt stress, these genes should play a key role in protein biosynthesis in roots of sweet sorghum. In contrast, almost no DEGs associated with cell membranes were identified in leaf blades of sweet sorghum after NaCl treatment for 6 and 48 h (Figures S6 and S7), suggesting that this salt treatment might not severely affect the membrane stability in leaf blades of sweet sorghum.

Photosynthesis is a vital process of primary metabolism and provides a large extent of energy and carbohydrates for plant growth and development [50]. However, the photosynthesis of most plant species is generally inhibited under saline conditions as a consequence of lessened CO_2_ availability due to stomatal closure, disturbed chloroplast light energy capture, hindered photosynthetic electron flow and carbon assimilation capacity [51]. Differently, it has been reported that the photosynthesis rate, stomatal pore size and PSII photochemical efficiency of a salt-tolerant sweet sorghum cultivar are all maintained at high levels under NaCl treatments [11]. In the present study, it was found that sweet sorghum cultivar “Lvjuren” showed a high salt tolerance, as its growth was unaffected by 50 and 100 mM NaCl treatments (Figure 1). Furthermore, the net photosynthesis rate and stomatal conductance of “Lvjuren” under 50 and 100 mM NaCl treatments were maintained as stable, and chlorophyll b content was unaffected when external NaCl concentration was up to 200 mM (Figure 2). All these results suggested that sweet sorghum possesses a strong photosynthetic ability under saline environments.

The cultivation of sweet sorghum in large-scale salinized areas is thought to be a promising approach to ensure food security and promote ecological restoration [2,3]. For this purpose, the strong photosynthetic ability of sweet sorghum under saline environments could (i) provide large amounts of resources (leaves and stalks) for producing silage and hay; (ii) develop roots and shoots for sand fixation and soil reservation; and (iii) accumulate sugars for energy production. Researchers have analyzed the expression changes of genes involved in photosynthetic processes such as chlorophyll biosynthesis, carbon fixation, photosystem I and II and sugar biosynthesis of sweet sorghum under NaCl treatments using transcriptome sequencing [11]. In the present study, GO analysis indicated that after NaCl treatment for both 6 and 48 h, the majority of DEGs involved in cellular components in leaf blades were categorized into chloroplasts, such as chloroplast stroma, chloroplast envelope, chloroplast thylakoid membrane and thylakoid (Figures S6 and S7). Therefore, sweet sorghum should have evolved an excellent ability to maintain the function of chloroplasts to sustain photosynthetic performance and sugar biosynthesis, which provides an important theoretical basis for the cultivation of this species in marginal lands.

## 4. Materials and Methods

### 4.1. Plant Material and Growth Conditions

Seeds of “Lvjuren”, a sweet sorghum cultivar in China, were obtained from Beijing Best Grass Industry Co., Ltd. (Beijing, China). The seeds were sterilized in 75% ethanol for 5 min, rinsed in distilled water 3 times and then sown in 0.5 L plastic pots (4–5 seeds per pot) filled with coarse silica sand with a particle diameter of about 0.5 cm. All pots were placed in trays (20 pots per tray), and 2 L modified Hoagland solution (5 mM KNO_3_, 1 mM KH_2_PO_4_, 1 mM MgSO_4_, 1 mM Ca(NO_3_)_2_, 60 μM Fe-citrate, 50 μM H_3_BO_3_, 10 μM MnCl_2_, 1.6 μM ZnSO_4_, 0.6 μM CuSO_4_ and 0.05 μM Na_2_MoO_4_, pH = 5.7) was added into trays_._ The Hoagland solution was renewed every 3 d. The growth conditions were as follows: a constant temperature of 28 °C, 16 h light period with the light flux density of approximately 500 mmol/m^2^/s and relative humidity of approximately 60–80%.

After 3 weeks, the seedlings were thinned out to 1 uniform plant in each pot. Then, 2 L Hoagland solution supplemented with 0 (control), 50, 100 or 200 mM NaCl was added into trays. The 100 and 200 mM NaCl treatments were increased by 50 mM each day until final concentrations were achieved to avoid salt shock. All solutions were changed every 2 d to maintain a constant NaCl concentration. After 10 d, seedlings were harvested for the measurement of physiological parameters. Six replicate seedlings were used for all measurements (*n* = 6).

### 4.2. Determination of Plant Height, Tissue Biomass and Shoot Water Content

The plant height (PH) of individual seedlings was measured first; then, the root and shoot were separated, and fresh weight (FW) was measured. All samples were finally oven-dried at 80 °C for 3 d to measure dry weight (DW). The shoot water content was calculated as (FW-DW)/DW [30].

### 4.3. Measurements of Photosynthesis-Related Parameters

The leaf net photosynthesis rate (Pn) and stomatal conductance (Gs) were measured with the LI-6400 photosynthetic measuring apparatus (LI-COR Biosciences, Lincoln, NE, USA) according to Ma et al. [34]. The chlorophyll in leaf samples was extracted with 80% acetone and 95% ethanol (1:1, *v*/*v*) in the dark for 24 h; then, after centrifugation, the absorbances at 645 nm and 663 nm of supernatant were measured using a UV spectrophotometer (UV-2102C, Unico Instrument Co., Ltd., Shanghai, China). The chlorophyll a and b contents were calculated using the following formula: Chla content (mg/g FW) = (12.71 × OD_663_ − 2.69 × OD_645_) × V/(1000 × W), Chlb content (mg/g FW) = (22.9 × OD_645_ − 4.68 × OD_663_) × V/(1000 × W), where V refers to the volume of extraction solution (10 mL) and W refers to the fresh weight of leaf samples [52].

### 4.4. Measurement of Ion Contents in Tissues

The root, leaf sheath and leaf blade samples of seedlings were first put in a 120 °C oven for 20 min then thoroughly dried at 80 °C. After that, samples were incubated in 100 mM acetic acid at 90 °C for 2 h, then Na^+^ and K^+^ contents in tissues were determined using a flame spectrophotometer (Model 410 Flame; Sherwood Scientific, Ltd., Cambridge, UK) according to Wang et al. [53]. Samples were also incubated with deionized water at 100 °C for 2 h, then tissue Cl^−^ content was determined using a chloride analyzer (Model 926, Sherwood Scientific Ltd., Cambridge, UK) [40], and tissue NO_3_^−^ content was determined by the colorimetric method as described by Drechsler et al. [54].

### 4.5. Transcriptome Sequencing

For transcriptome sequencing, the seedlings of sweet sorghum cultivar “Lvjuren” were cultured as described in Section 4.1. After 3 weeks, uniform seedlings were divided into the control (C) group and salt treatment (S) group. In the C group, seedlings were irrigated with Hoagland solution for 6 and 48 h, then root (R), leaf sheath (LS) and leaf blade (LB) samples were collected and labeled as C6R, C6LS, C6LB, C48R, C48LS and C48LB. In the S group, seedlings were treated with 200 mM NaCl for 6 and 48 h, then root, leaf sheath and leaf blade samples were collected and labeled as S6R, S6LS, S6LB, S48R, S48LS and S48LB. Each sample had three biological replicates (*n* = 3).

Total RNA was extracted from above samples and then converted into mRNA sequencing libraries using the Illumina HiSeq platform (Biomarker Technologies Co., Ltd., Beijing, China). Therefore, a total of 36 independent libraries were sequenced. The raw reads were filtered by removal of the adaptor sequences and low-quality sequence reads (containing poly-N) to obtain high-quality clean reads. Subsequently, the clean reads were mapped to the *Sorghum bicolor* reference genome sequence (NCBI accession number: GCF_000003195.3, accessed on 7 April 2017, https://www.ncbi.nlm.nih.gov/genome/?term=sorghum). The reads with a perfect match or one mismatch were further analyzed and annotated based on the reference genome. Finally, the gene function was annotated by aligning against protein databases including Nr, Nt, Pfam, KOG/COG, Swiss-Prot, KO and GO.

### 4.6. Differentially Expressed Genes Analysis

Gene expression levels in each library were determined by the fragments per kilobase of transcript per million fragments mapped (FPKM) method as described by Mortazavi et al. [55]. Differential expression analysis of each treatment group against the corresponding control group (e.g., C6R-1, C6R-2, C6R-3 vs. S6R-1, S6R-2, S6R-3) was performed using the DESeq2 software 1.30.1 [56]. The resulting *p* values were adjusted using the Benjamini and Hochberg’s approach for controlling the false discovery rate (FDR) [57]. In this study, a gene with FDR < 0.001 and absolute value of log_2_(FPKM_treated_/FPKM_control_) > 1 was termed as a differentially expressed gene (DEG). Finally, we analyzed DEGs related to Na^+^, K^+^, Cl^−^ and NO_3_^−^ transport after salt treatment for 6 and 48 h, and screened DEGs encoding transcription factors after salt treatment for 6 h.

Cellular organelles are crucial for stress tolerance of plants [10]. To identify key genes that are possibly involved in maintaining cell organelle characteristics, we at last performed GO analysis on DEGs that are categorized into cellular components in different tissues after salt treatment for 6 and 48 h.

### 4.7. Validation of RNA-Sequencing Results

To confirm the reliability of RNA sequencing (RNA-seq) results, we randomly selected 20 genes in transcriptome data and determined the relative expression levels of these genes using the qRT-PCR method with a StepOnePlus Real-Time PCR Thermocycler (ABI PRISM 7500, Applied Biosystems, Foster City, CA, USA) [40]. Sweet sorghum internal reference gene *SbActin1* was used as a control. The gene-specific primers for qRT-PCR are listed in Table S1. Finally, the correlation analysis between RNA-seq and qRT-PCR results was performed.

### 4.8. Analysis of Expression Pattern of HKT1;5, CLCc and NPF7.3-1 in Sweet Sorghum under NaCl Treatment

Sweet sorghum showed strong abilities to restrict Na^+^ accumulation in shoots, reserving large amounts of Cl^−^ in leaf sheaths and maintaining high NO_3_^−^ content in leaf blades (Figure 2). HKT1;5 has been reported to play a key role in restricting the long-distance transport of Na^+^ from roots into shoots of rice [17]. CLCc has been thought to facilitate Cl^−^ accumulation in plant tissues by mediating vacuolar Cl^−^ compartmentalization [39], and NPF7.3 (also named as NRT1.5) helps the transport of NO_3_^−^ into shoots by mediating the efflux of NO_3_^−^ from root stele to xylem sap [46]. As our transcriptome data showed that the expression of *HKT1;5* in roots of sweet sorghum was upregulated after NaCl treatment for 6 h (Table S5) and the expression of *CLCc* and *NPF7.3-1* in roots and leaf sheaths of sweet sorghum was also upregulated after salt treatment for both 6 and 48 h (Figure 7), we analyzed in detail the expression pattern of these three genes in response to 200 mM NaCl treatment using the qRT-PCR method.

The 3-week-old sweet sorghum seedlings were cultured as described in Section 4.1, then uniform seedlings were treated with 200 mM NaCl for 0, 3, 6, 24, 48 and 72 hRoot, leaf sheath and leaf blade samples were harvested, the total RNA in these samples was extracted and the first-strand cDNA was synthesized using the PrimeScript™ RT Master Mix (Perfect Real Time) kit (TaKaRa, Biotech Co., Ltd., Dalian, China). The relative expression levels of these genes in above samples were determined by the qRT-PCR method according to Duan et al. [58]. The primers for *HKT1;5*, *CLCc* and *NPF7.3-1* are listed in Table S1.

### 4.9. Data Analysis

Six replicate seedlings were used for all physiological parameter measurements (*n* = 6), and three replicate seedlings were used for transcriptome sequencing and qRT-PCR analysis (*n* = 3). The results for the physiological parameters and qRT-PCR are all presented as the mean with standard deviation (SD). The data were subjected to one-way analysis of variance (ANOVA) using SPSS statistical software 19.0 (SPSS Inc., Chicago, IL, USA) followed by Tukey’s HSD to detect significant differences between means at a significance level of *p* < 0.05.

## 5. Conclusions

In conclusion, our results demonstrated that sweet sorghum is a typical Na^+^ exclusion plant species that can maintain a low Na^+^ content in shoots under salt stress. Although sweet sorghum cannot restrict Cl^−^ translocation into shoots, it decreases Cl^−^ toxicity to leaf blades by the large accumulation of Cl^−^ in leaf sheaths. Furthermore, sweet sorghum shows a prominent ability for maintaining NO_3_^−^ homeostasis in leaf blades under NaCl treatments. Transcriptome sequencing identified many key genes involved in Na^+^, K^+^, Cl^−^ and NO_3_^−^ transport in roots, leaf sheaths and leaf blades of sweet sorghum after NaCl treatment. Furthermore, the increased expressions of *HKT1;5*, *CLCc* and *NPF7.3-1* after salt treatment are conducive to retention of Na^+^ in roots, accumulation of Cl^−^ in leaf sheaths and maintaining a high NO_3_^−^ content in leaf blades, respectively. Many *TFs* also play essential regulatory roles in the salt tolerance of sweet sorghum. In addition, sweet sorghum possesses a strong ability to maintain membrane stability and photosynthetic performance under salt stress. Further studies on the function of genes identified in the present work would help to comprehensively uncover adaptative mechanisms of sweet sorghum to saline environments and provide theoretical basis for the large-scale cultivation of sweet sorghum in salinized areas to ensure food security and promote ecological restoration.

## Data Availability

The clean sequencing data have been uploaded to the NCBI Sequence Read Archive (SRA) under the accession number PRJNA977880, the data that support the findings of this study are available from the corresponding author upon reasonable request.

## Supplementary Materials

The following supporting information can be downloaded at: https://www.mdpi.com/article/10.3390/ijms241311045/s1.

## Abbreviations

NHX: sodium/hydrogen exchanger; CHX: cation/H^+^ antiporter; CCX: cation/calcium exchanger; NCX: sodium/calcium exchanger; CNGC: cyclic nucleotide-gated ion channel; HKT: high affinity K^+^ transporter; KEA: K^+^ efflux antiporter; KT/KUP/HAK: potassium transporter; AKT: inward rectifying K^+^ channel; KOR: outward rectifying K^+^ channel; CLC: chloride channel; SLAH: slow-type anion channel-associated homolog; ALMT: aluminum-activated malate transporter; NPF: nitrate transporter1/peptide transporter; CCC: cation chloride cotransporter; WRKY: WRKY DNA-binding domain transcription factor; MYB: MYB-like DNA-binding transcription factor; NAC: NAC domain-containing transcription factor; bHLH: helix-loop-helix DNA-binding domain transcription factor; AP2: AP2 domain transcription factor; ERF: ethylene-responsive transcription factor; bZIP: basic leucine-zipper transcription factor; MADS-box: MADS-box transcription factor; HSF: heat stress transcription factor; ZF: zinc finger; GRAS: GRAS domain transcription factor; Nr: NCBI non-redundant protein sequences; Nt: NCBI non-redundant nucleotide sequences; Pfam: protein family; KOG/COG: clusters of orthologous groups of proteins; Swiss-Prot: a manually annotated and reviewed protein sequence database; KO: KEGG ortholog database; GO: gene ontology.
