# Supplementary material for: Physiological and Transcriptional Analyses Provide Insight into Maintaining Ion Homeostasis of Sweet Sorghum under Salt Stress
Source: Int J Mol Sci. 2023 Jul 3;24(13):11045. doi: 10.3390/ijms241311045 (PMC10341859; doi:10.3390/ijms241311045)
Supplement: Supplementary file 1 [file ijms-24-11045-s001.zip › ijms-2455843-supplementary.pdf]

Table S1 The primers for qRT-PCR analysis.

Gene ID	Forward primers (5′-3′)	Reserve primers (5′-3′)
gene-LOC8085162	CTGCTTGGAGAGAACCTTGGAT	GCTTCCTTTCCTTCCTTTGC
gene-LOC8082181	CCATAAAACGAACGGTCAAGT	TTTCTGTCCTACATCCAACTCACT
gene-LOC8078430	AGAGTATGCTTTGCTATGTCGG	GAGCACCATCCTTTATTACCCA
gene-LOC8058543	TGGAGAAGCAAACATTGGTGA	TAGTGGCGGAATCTCGTAGC
gene-LOC8056614	GAGTGAAGCCAATGCTGTTCG	CTTGTTCCTCGTCGTTGCC
gene-LOC8055227	TGAAGCACCTGCCAAACG	TCCTCACTGAACCTCACCTCC
gene-LOC8070790	AAGGCTGGTGGTGCTTACAC	GAACCCCAATATGCCGAAC
gene-LOC8084948	TGGCTGATTGATTTGCTGG	CTGTTCGATCTTTGCTTTGACA
gene-LOC8059789	TGAGACCTCGAATACAAAAGCC	GGGAGTAGTGGTGGATGGAG
gene-LOC8076648	ACTCGGATCAGTCAAGAACAACT	CACAAGAAACCCCAAAAGAACA
gene-LOC8055234	TGCTTAGCGACGACGAGTTA	CAAGCTGACGGTTTGTTGG
gene-LOC110429509	TGGAGCAGTGGTATCCGTAGT	CCTTAGCATCTTCCTCGTCGTT
Sorghum_bicolor_newGene_8048	CCACGTAGCGTTCATACAATACA	GAACCTCTTGAGCGACACCTT
gene-LOC8079914	ACGCAGCATTAGTTGATTAGTGTC	GGCTGCTGTTGGAGGTGTT
gene-LOC8054416	CTCTTGATGCTCGGGCTTAT	TACACGTTTTACCTTCATCTCCTC
Sorghum_bicolor_newGene_1242	AGCAGGTGGTTCATGTTGGG	TGGCATTGGACACGGAAGA
gene-LOC8065743	CGTCCCGTTGTCTGCTTTC	GCTTTCCCTTTTGGATGTTCTT
gene-LOC8071420	AGGATGACTTATGCGTTCTCG	TCGGCACTTCTTGCTTGTTA
gene-LOC8061392	CCAGATCGGAAGGCAAGAG	AGGTGGTGGTGTTAGGATGAAC
gene-LOC8077869	CCCTCGGAGTAGAACTGGTATC	GTTGGTATGAGTGGCATGTGAA
SbActin1	ACGGCCTGGATGGCGACGTACATG	CAGAAGGACGCCTACGTTGGTGA
SbHKT1;5	CAAAATGAGGCACAATCCG	TGAAGATGGTTAGGCACGAGA
SbCLCc	TCTTAGCTCAACCGTCCCG	CCCGAATACCCCACCAATT
SbNPF7.3-1	TCTTGTTGGTGAAGCCCTCC	TAGGTGGACAGGTAGAACAGCG

Table S2 RNA sequencing production statistics.

Samples	Clean reads	Clean bases	GC content	%≥Q30
C6R-1	21,685,513	6,492,025,670	51.84%	90.19%
C6R-2	21,233,187	6,353,745,880	52.20%	91.58%
C6R-3	21,606,754	6,467,788,398	52.10%	91.27%
S6R-1	21,515,441	6,439,315,572	52.62%	90.32%
S6R-2	20,097,384	6,012,174,714	52.35%	90.52%
S6R-3	20,990,964	6,284,440,252	52.42%	89.99%
C6LS-1	28,104,739	8,414,582,254	54.33%	89.85%
C6LS-2	20,845,787	6,237,048,094	54.64%	90.81%
C6LS-3	21,969,154	6,574,479,452	53.65%	90.58%
S6LS-1	28,741,639	8,600,456,600	54.81%	90.54%
S6LS-2	22,028,872	6,592,878,370	54.47%	91.47%
S6LS-3	26,194,256	7,841,811,600	54.72%	90.10%
C6LB-1	20,158,254	6,032,714,478	53.44%	91.86%
C6LB-2	21,992,758	6,581,387,904	54.49%	90.62%
C6LB-3	27,775,529	8,313,354,526	55.79%	87.87%
S6LB-1	21,024,819	6,292,190,276	53.52%	90.58%
S6LB-2	21,528,712	6,442,747,000	53.92%	90.38%
S6LB-3	21,783,002	6,519,895,574	55.13%	90.43%
C48R-1	24,884,696	7,449,241,432	51.72%	91.03%
C48R-2	23,771,732	7,118,292,168	51.50%	91.54%
C48R-3	20,918,074	6,258,920,318	52.37%	92.44%
S48R-1	27,922,513	8,359,596,758	52.69%	89.56%
S48R-2	24,897,142	7,451,136,196	52.21%	91.13%
S48R-3	28,331,032	8,479,638,056	52.01%	91.13%
C48LS-1	25,869,891	7,741,653,466	53.17%	90.42%
C48LS-2	25,190,278	7,541,864,158	54.06%	89.83%
C48LS-3	25,412,707	7,606,135,144	54.14%	90.51%
S48LS-1	23,437,249	7,012,940,050	53.70%	90.69%
S48LS-2	28,746,121	8,604,160,636	54.23%	90.10%
S48LS-3	23,830,152	7,136,317,078	53.39%	89.27%
C48LB-1	26,155,517	7,823,294,822	53.15%	94.27%
C48LB-2	21,980,635	6,578,056,582	54.15%	94.35%
C48LB-3	25,056,506	7,492,470,902	54.58%	94.26%
S48LB-1	29,157,844	8,726,376,028	52.54%	94.65%
S48LB-2	21,907,906	6,557,487,796	53.25%	94.31%
S48LB-3	29,696,308	8,884,796,634	52.62%	95.17%

Note: Sweet sorghum seedlings were treated with Hoagland solution (C) and 200 mM NaCl (S) for 6 and 48 h, then root (R), leaf sheath (LS) and leaf blades (LB) were harvested for RNA sequencing.

Table S3 The statistics of results by mapping the RNA-seq data to sorghum reference genome sequence

Samples	Total reads	Mapped reads	Reads map to "+"	Reads map to "-"
C6R-1	43,371,026	38,069,628 (87.78%)	19,710,682 (45.45%)	19,898,943 (45.88%)
C6R-2	42,466,374	37,845,248 (89.12%)	19,447,547 (45.80%)	19,615,511 (46.19%)
C6R-3	43,213,508	38,374,997 (88.80%)	19,849,744 (45.93%)	20,019,019 (46.33%)
S6R-1	43,030,882	37,324,778 (86.74%)	19,576,281 (45.49%)	19,757,452 (45.91%)
S6R-2	40,194,768	34,485,560 (85.80%)	18,103,839 (45.04%)	18,265,626 (45.44%)
S6R-3	41,981,928	35,791,386 (85.25%)	19,030,194 (45.33%)	19,197,395 (45.73%)
C6LS-1	56,209,478	49,344,015 (87.79%)	25,439,033 (45.26%)	25,611,101 (45.56%)
C6LS-2	41,691,574	36,359,794 (87.21%)	19,055,329 (45.71%)	19,153,020 (45.94%)
C6LS-3	43,938,308	38,477,728 (87.57%)	19,882,229 (45.25%)	20,036,908 (45.60%)
S6LS-1	57,483,278	50,439,106 (87.75%)	26,012,219 (45.25%)	26,146,753 (45.49%)
S6LS-2	44,057,744	38,981,598 (88.48%)	20,154,201 (45.74%)	20,248,097 (45.96%)
S6LS-3	52,388,512	45,572,110 (86.99%)	23,605,340 (45.06%)	23,765,759 (45.36%)
C6LB-1	40,316,508	35,352,759 (87.69%)	18,540,209 (45.99%)	18,626,006 (46.20%)
C6LB-2	43,985,516	38,845,218 (88.31%)	20,053,183 (45.59%)	20,173,413 (45.86%)
C6LB-3	55,551,058	45,757,244 (82.37%)	24,581,265 (44.25%)	24,890,528 (44.81%)
S6LB-1	42,049,638	36,797,978 (87.51%)	18,998,626 (45.18%)	19,181,437 (45.62%)
S6LB-2	43,057,424	37,731,052 (87.63%)	19,523,975 (45.34%)	19,683,816 (45.72%)
S6LB-3	43,566,004	38,128,602 (87.52%)	19,926,958 (45.74%)	20,069,397 (46.07%)
C48R-1	49,769,392	44,051,546 (88.51%)	22,680,304 (45.57%)	22,877,198 (45.97%)
C48R-2	47,543,464	42,162,668 (88.68%)	21,741,573 (45.73%)	21,902,460 (46.07%)
C48R-3	41,836,148	37,658,794 (90.01%)	19,424,935 (46.43%)	19,553,847 (46.74%)
S48R-1	55,845,026	47,337,035 (84.76%)	24,284,167 (43.48%)	24,555,073 (43.97%)
S48R-2	49,794,284	44,037,992 (88.44%)	22,642,492 (45.47%)	22,830,208 (45.85%)
S48R-3	56,662,064	50,213,260 (88.62%)	25,824,759 (45.58%)	26,036,372 (45.95%)
C48LS-1	51,739,782	45,315,969 (87.58%)	23,387,619 (45.20%)	23,595,282 (45.60%)
C48LS-2	50,380,556	44,088,539 (87.51%)	22,753,909 (45.16%)	22,951,860 (45.56%)
C48LS-3	50,825,414	44,646,440 (87.84%)	23,045,305 (45.34%)	23,201,756 (45.65%)
S48LS-1	46,874,498	41,348,439 (88.21%)	21,304,418 (45.45%)	21,448,462 (45.76%)
S48LS-2	57,492,242	50,531,900 (87.89%)	26,084,986 (45.37%)	26,293,729 (45.73%)
S48LS-3	47,660,304	41,725,172 (87.55%)	21,344,824 (44.79%)	21,547,989 (45.21%)
C48LB-1	52,311,034	47,294,936 (90.41%)	24,827,121 (47.46%)	24,794,296 (47.40%)
C48LB-2	43,961,270	40,676,282 (92.53%)	21,118,062 (48.04%)	21,089,411 (47.97%)
C48LB-3	50,113,012	45,969,091 (91.73%)	24,047,689 (47.99%)	23,994,421 (47.88%)
S48LB-1	58,315,688	53,739,643 (92.15%)	27,852,391 (47.76%)	27,859,498 (47.77%)
S48LB-2	43,815,812	40,215,598 (91.78%)	21,143,529 (48.26%)	21,138,233 (48.24%)
S48LB-3	59,392,616	52,779,798 (88.87%)	28,606,642 (48.17%)	28,605,605 (48.16%)

Note: A public genome data (NCBI accession number: GCF_000003195.3) was used as the reference genome sequence.

Table S4 Functional annotation of new genes that cannot map to reference genome sequence

Annotated databases	New gene number
COG	190
GO	1,147
KEGG	680
KOG	432
Pfam	763
Swiss-Prot	513
TrEMBL	2,412
eggNOG	1,020
nr	2,477
All	2,574

Table S5 The expression change of DEGs related to ion transport in roots of sweet sorghum after 200 mM NaCl treatment for 6 h

Gene ID	Log_2 value	Annotation
gene-LOC8074408	1.642605	NHX2
gene-LOC8084417	3.403835	CHX19-1
gene-LOC8084418	2.65564	CHX19-2
gene-LOC8057212	3.068639	CCX-1
gene-LOC8071496	2.004107	CCX-2
gene-LOC110434083	1.117284	NCX1
gene-LOC8081919	1.273911	CNGC20
gene-LOC8078183	1.606078	HKT1;5
gene-LOC8076484	-1.00874	KEA2
gene-LOC8071480	-1.48248	KEA3
gene-LOC8072046	2.208412	HAK1
gene-LOC8067862	2.527079	HAK5-1
gen-LOC8078559	1.486856	HAK9
gene-LOC8063025	3.19092	HAK16
gene-LOC8062763	1.869305	HAK17
gene-LOC8065633	2.340472	HAK21
gene-LOC8079532	-1.49882	KUP5-1
gene-LOC8083092	-2.03063	KUP5-2
gene-LOC8061543	-1.20175	KUP8
gene-LOC8058492	1.213374	AKT2
gene-LOC8057743	-1.68167	AKT2/3
gene-LOC8075862	-2.35421	KOR2
gene-LOC8069763	1.650568	CLCa
gene-LOC8080343	2.792361	CLCc
gene-LOC8079456	-1.76868	CLCg
gene-LOC8077536	-1.4744	SLAH1
gene-LOC8079295	2.556683	SLAH2
gene-LOC8076556	-1.07108	SLAH3
gene-LOC8077536	-1.4744	SLAH4
gene-LOC8073008	3.450748	ALMT1
gene-LOC8065254	1.71191	NPF2.11
gene-LOC8085131	1.682452	NPF3.1-1
gene-LOC8061532	-1.35763	NPF3.1-2
gene-LOC8075842	1.903989	NPF4.5
gene-LOC8067440	-2.29952	NPF5.2
gene-LOC8075730	-1.99354	NPF5.9
gene-LOC8062396	-1.17969	NPF5.10

gene-LOC8067856	1.05965	NPF6.3
gene-LOC8070561	1.327672	NPF6.4
gene-LOC8071782	5.152358	NPF7.3-1
gene-LOC8073896	1.809284	NPF7.3-2
gene-LOC8062975	1.194403	P-H^+-ATPase
gene-LOC8072298	1.67756	P-Ca^{2+}-ATPase 5
gene-LOC8064910	2.918003	P-Ca^{2+}-ATPase 7
gene-LOC8081867	1.393817	P-Ca^{2+}-ATPase 10

Table S6 The expression change of DEGs related to ion transport in roots of sweet sorghum after 200 mM NaCl treatment for 48 h

Gene ID	Log_2 value	Annotation
gene-LOC8084417	2.252446	CHX19-1
gene-LOC8084418	1.737723	CHX19-2
gene-LOC8057212	1.482209	CCX-1
gene-LOC8071496	1.275421	CCX-2
gene-LOC8081919	1.054428	CNGC20
gene-LOC8076484	-1.08529	KEA2
gene-LOC8072092	3.079258	KT3
gene-LOC8063025	2.169547	HAK16
gene-LOC8065633	3.380665	HAK21
gene-LOC8081576	1.273335	HAK22
gene-LOC8065785	1.009076	HAK24
gene-LOC8057743	-1.74723	AKT2/3
gene-LOC8075862	-3.87659	KOR2
gene-LOC8080343	1.264192	CLCc
gene-LOC8079456	-1.9079	CLCg
gene-LOC8079295	2.208967	SLAH2
gene-LOC8066616	1.792982	SLAH3
gene-LOC8073008	2.230816	ALMT1
gene-LOC8065254	1.485028	NPF2.11
gene-LOC8061309	2.283274	NPF2.7
gene-LOC8085131	3.015571	NPF3.1-1
gene-LOC8061532	-2.19179	NPF3.1-2
gene-LOC8060182	1.335163	NPF4.3-1
gene-LOC8060184	1.069603	NPF4.3-2
gene-LOC8085518	-2.17604	NPF5.2
gene-LOC8055690	-1.16613	NPF5.6
gene-LOC8075730	-2.11996	NPF5.9
gene-LOC8063327	1.397916	NPF6.1
gene-LOC8058104	-1.97736	NPF6.3
gene-LOC8070561	1.190999	NPF6.4
gene-LOC8071782	3.871156	NPF7.3-1
gene-LOC8073896	2.932143	NPF7.3-2
gene-LOC110433327	1.897474	NPF8.1
gene-LOC8083625	2.170176	NPF8.3
gene-LOC8062975	1.096462	P-H^+-ATPase

Table S7 The expression change of DEGs related to ion transport in leaf sheaths of sweet sorghum after 200 mM NaCl treatment for 6 h

Gene ID	Log_2 value	Annotation
gene-LOC8070185	1.441058	NCX
gene-LOC8065479	-1.21121	NCX
gene-LOC8076497	-1.29114	NCX
gene-LOC8072309	2.33587	HKT1;4
gene-LOC8085586	-1.78833	KT1
gene-LOC8083518	1.906225	HAK4
gene-LOC8078559	1.06849	HAK9
gene-LOC8058236	1.270356	AKT1
gene-LOC8080343	1.379157	CLCc
gene-LOC8079230	1.610902	NPF1.2
gene-LOC8076953	2.827796	NPF3.1
gene-LOC8076954	-1.58151	NPF3.1
gene-LOC8084233	-3.31167	NPF4.3
gene-LOC8055194	1.519585	NPF5.10
gene-LOC8071782	1.625425	NPF7.3-1
gene-LOC8083627	-1.23059	NPF8.3
gene-LOC8055893	-1.41426	P-Ca^{2+}-ATPase 5
gene-LOC8081867	1.210822	P-Ca^{2+}-ATPase 10

Table S8 The expression change of DEGs related to ion transport in leaf sheaths of sweet sorghum after 200 mM NaCl treatment for 48 h

Gene ID	Log_2 value	Annotation
gene-LOC110433848	1.820332	NCX1a
gene-LOC8083092	-1.01034	KUP5
gene-LOC8062763	1.921104	HAK17
gene-LOC8080343	1.0635	CLCc
gene-LOC8076043	3.286561	ALMT10
gene-LOC8085131	3.551471	NPF3.1
gene-LOC8076953	2.222769	NPF3.1
Sorghum_bicolor_newGene_2569	-2.41239	NPF4.5
gene-LOC8071782	5.487174	NPF7.3-1
gene-LOC8073896	1.713527	NPF7.3-2
gene-LOC8067856	1.021939	NPF8.1
gene-LOC8079011	-1.17202	NPF8.3
gene-LOC8078037	-1.08319	P-H^+-ATPase
gene-LOC8067356	1.158969	P-Ca^{2+}-ATPase 4
gene-LOC8085626	1.258629	P-Ca^{2+}-ATPase 9
gene-LOC8081867	1.033328	P-Ca^{2+}-ATPase 10

Table S9 The expression change of DEGs related to ion transport in leaf blades of sweet sorghum after 200 mM NaCl treatment for 6 h

Gene ID	Log_2 value	Annotation
gene-LOC8063860	-1.27592	NHX1
gene-LOC8074408	-1.08542	NHX2
gene-LOC8067369	1.323903	CCX1
gene-LOC8062242	-1.70832	NCX
gene-LOC8056537	1.767412	NCX
gene-LOC110433848	1.150777	NCX1a
gene-LOC8072046	1.996546	HAK1
gene-LOC8067862	2.603512	HAK5-1
gene-LOC8067863	1.637198	HAK5-2
gene-LOC8085154	1.222118	HAK12
gene-LOC8080343	1.641406	CLCc
gene-LOC8071767	1.079116	CLCf
gene-LOC8069523	-1.28811	ALMT12
gene-LOC8079230	2.105721	NPF1.2
gene-LOC8076956	-1.66676	NPF3.1
gene-LOC8084233	-2.51835	NPF4.3
gene-LOC8067439	1.568329	NPF5.2
gene-LOC8062396	-2.9792	NPF5.10
gene-LOC8067856	1.364558	NPF6.3
gene-LOC110433833	2.216318	NPF8.1
gene-LOC8073241	-1.32138	P-H^+-ATPase
gene-LOC8076135	1.389746	P-Ca^{2+}-ATPase 5
gene-LOC8063993	1.364938	P-Ca^{2+}-ATPase 5
gene-LOC8064910	2.451387	P-Ca^{2+}-ATPase 7
gene-LOC8081867	2.365042	P-Ca^{2+}-ATPase 10

Table S10 The expression change of DEGs related to ion transport in leaf blades of sweet sorghum after 200 mM NaCl treatment for 48 h

Gene ID	Log_2 value	Annotation
gene-LOC8063860	-1.34885	NHX1
gene-LOC8055510	1.804957	CHX15
gene-LOC8056257	1.357069	NCX
gene-LOC110432929	1.000864	NCX
gene-LOC8062242	-1.05495	NCX
gene-LOC8056537	1.413177	NCX
gene-LOC8059087	-1.2577	NCX1c
gene-LOC8076484	-1.22532	KEA2
gene-LOC8071480	-1.12452	KEA3
gene-LOC8085586	-1.13764	KT1
gene-LOC8083092	-1.5998	KUP5
gene-LOC8077695	1.501153	KUP8-1
gene-LOC8061543	1.206681	KUP8-2
gene-LOC8065636	-1.43424	KUP16
gene-LOC8085154	1.876346	HAK12
gene-LOC8064024	-1.55661	HAK26
gene-LOC8057743	2.329447	AKT2/3
gene-LOC8058450	-2.1612	CLCa
gene-LOC8069763	-1.27955	CLCe
gene-LOC8071767	1.408988	CLCf
gene-LOC8068475	1.465604	CLCg-1
gene-LOC8079456	-1.11092	CLCg-2
gene-LOC8054905	1.232952	SLAH3
gene-LOC8073008	1.602577	ALMT1
gene-LOC8069523	-1.24231	ALMT12
gene-LOC8076953	3.940685	NPF3.1
gene-LOC8076956	-1.48041	NPF3.1
gene-LOC8085131	3.168706	NPF3.1
gene-LOC8084233	-2.31023	NPF4.3
gene-LOC8075842	-1.3223	NPF4.4
gene-LOC8082863	-2.22075	NPF4.6
gene-LOC8067440	-1.69068	NPF5.2
gene-LOC8062396	-2.55687	NPF5.10
gene-LOC8055194	3.922302	NPF5.10
gene-LOC8063327	1.047337	NPF6.1
gene-LOC8059535	-2.80299	NPF6.3
gene-LOC8071782	8.105068	NPF7.3-1
gene-LOC8073896	1.585449	NPF7.3-2
gene-LOC110433327	-1.44379	NPF8.1
gene-LOC8081572	-1.43396	NPF8.2
gene-LOC8079011	-1.62145	NPF8.3

gene-LOC8083625	2.037393	NPF8.3
gene-LOC8083636	-1.19868	NPF8.5
gene-LOC8072876	-1.58097	P-H^{+}-ATPase 1
gene-LOC8064910	2.601286	P-Ca^{2+}-ATPase 7
gene-LOC8085626	-1.36278	P-Ca^{2+}-ATPase 9

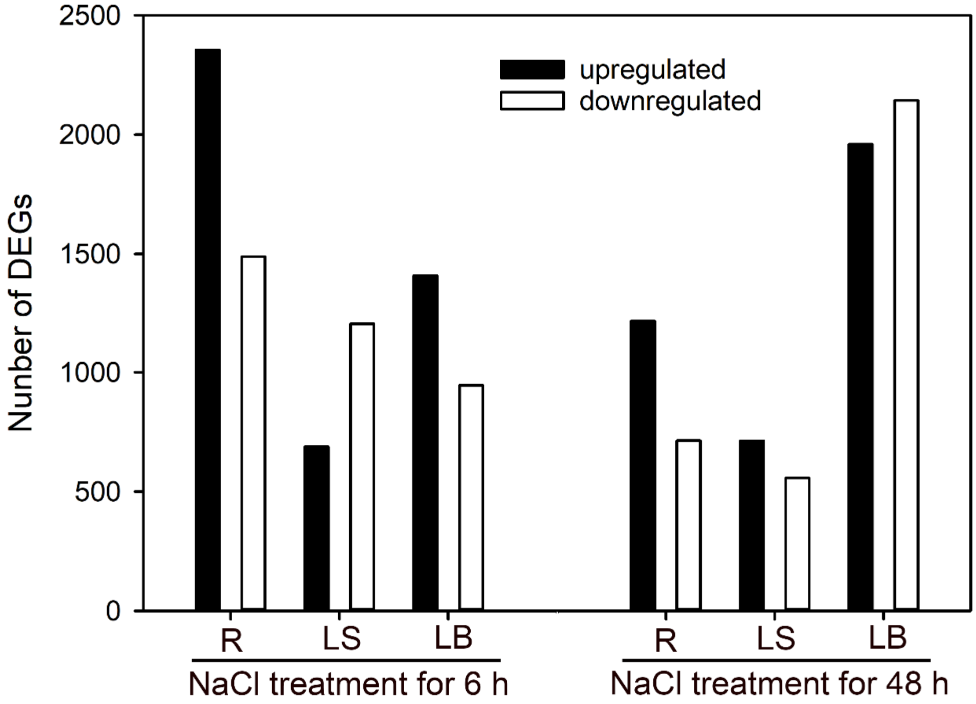

Figure S1 The number of DEGs in roots (R), leaf sheath (LS) and leaf blades (LB) after 200 mM NaCl treatment for 6 and 48 h.

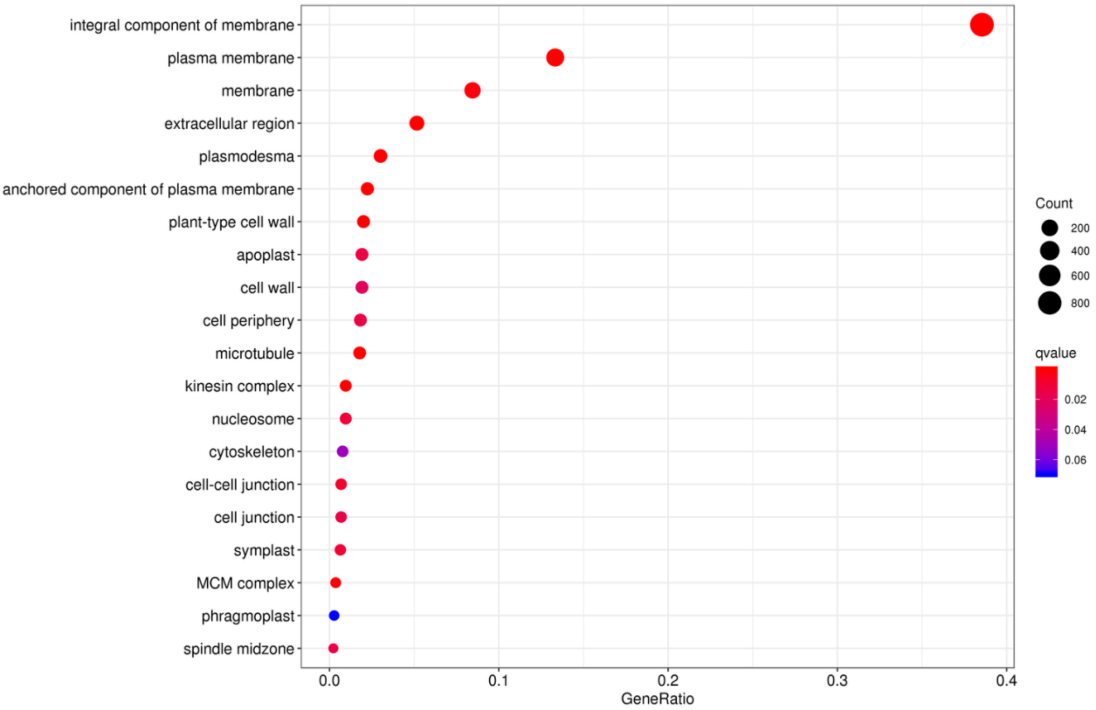

Figure S2 GO analysis on DEGs involved in cellular component in roots of sweet sorghum after 200 mM NaCl treatment for 6 h. The X axis indicates the percentage of GO termed genes among all DEGs, the Y axis indicated GO terms. The size of dots reflects the number of genes, the color of dots reflects the *q* value of hypergeometric test.

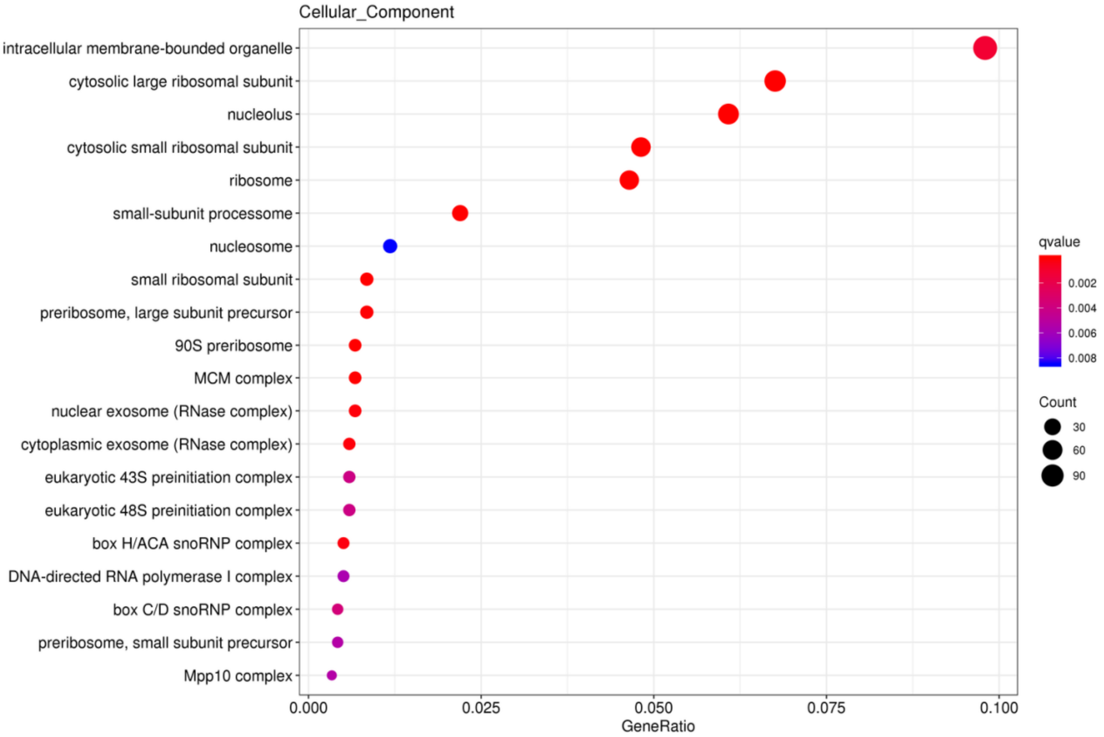

Figure S3 GO analysis on DEGs involved in cellular component in roots of sweet sorghum after 200 mM NaCl treatment for 48 h. The X axis indicates the percentage of GO termed genes among all DEGs, the Y axis indicated GO terms. The size of dots reflects the number of genes, the color of dots reflects the *q* value of hypergeometric test.

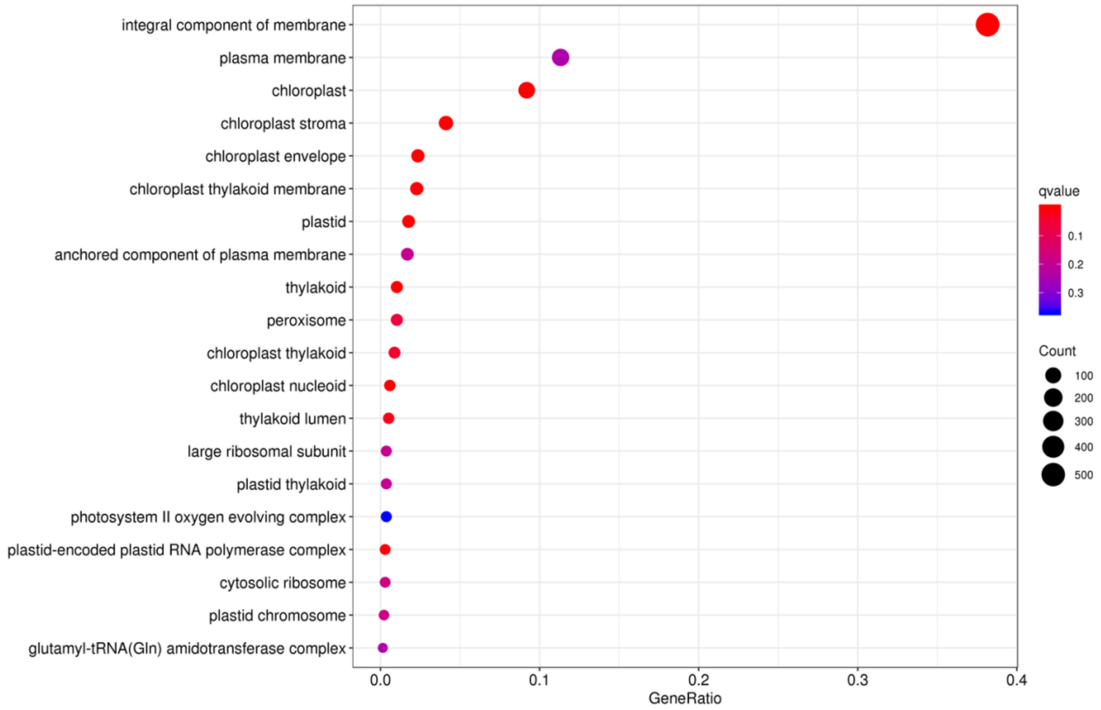

Figure S4 GO analysis on DEGs involved in cellular component in leaf sheaths of sweet sorghum after 200 mM NaCl treatment for 6 h. The X axis indicates the percentage of GO termed genes among all DEGs, the Y axis indicated GO terms. The size of dots reflects the number of genes, the color of dots reflects the *q* value of hypergeometric test.

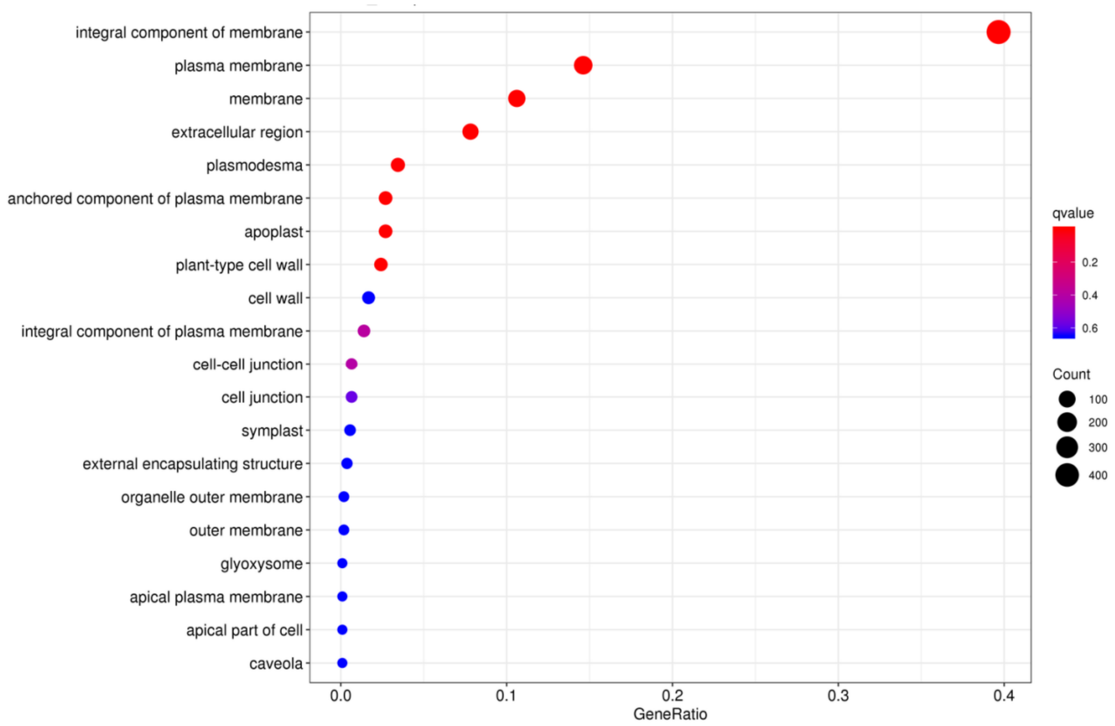

Figure S5 GO analysis on DEGs involved in cellular component in leaf sheaths of sweet sorghum after 200 mM NaCl treatment for 48 h. The X axis indicates the percentage of GO termed genes among all DEGs, the Y axis indicated GO terms. The size of dots reflects the number of genes, the color of dots reflects the *q* value of hypergeometric test.

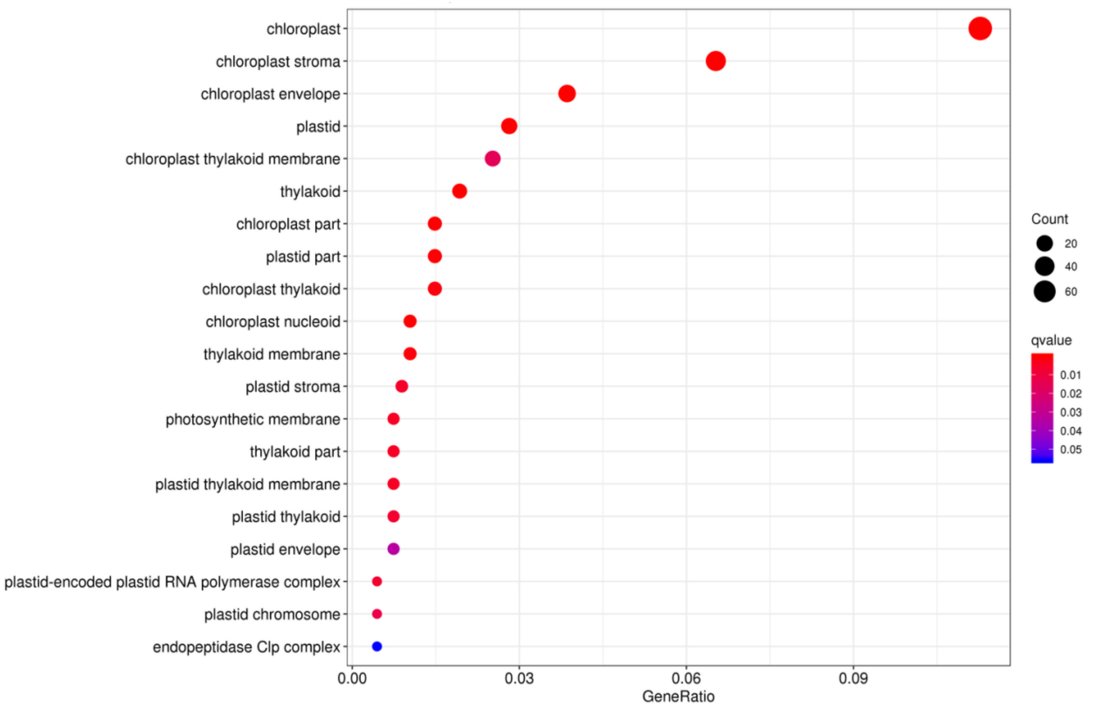

Figure S6 GO analysis on DEGs involved in cellular component in leaf blades of sweet sorghum after 200 mM NaCl treatment for 6 h. The X axis indicates the percentage of GO termed genes among all DEGs, the Y axis indicated GO terms. The size of dots reflects the number of genes, the color of dots reflects the *q* value of hypergeometric test.

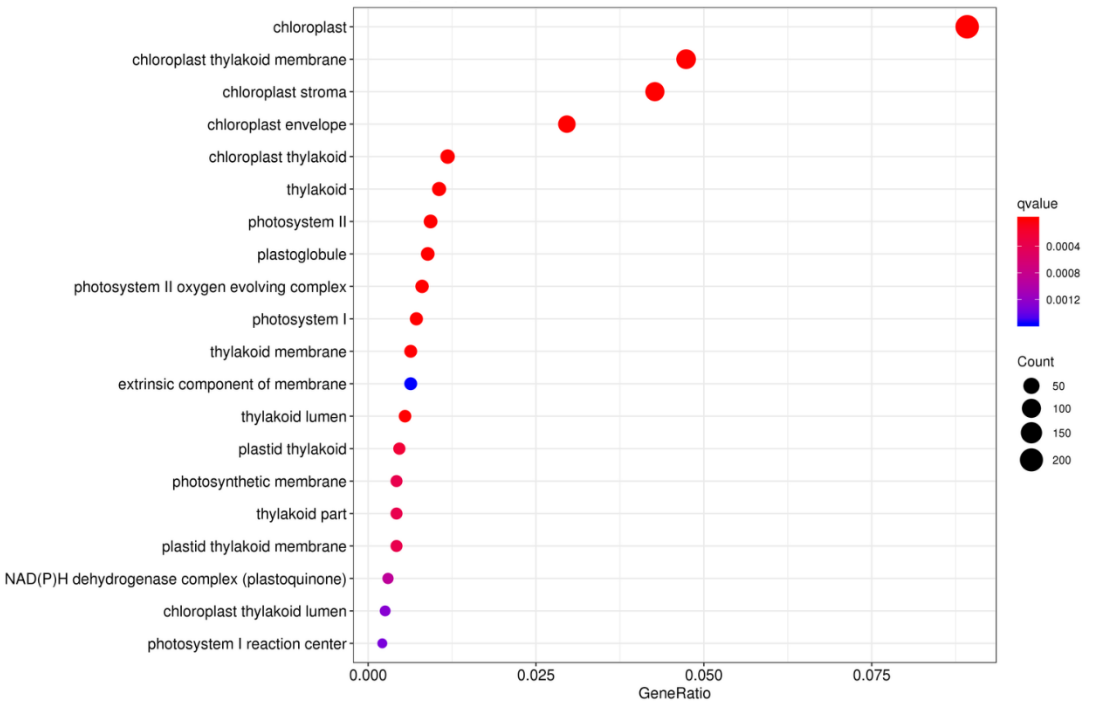

Figure S7 GO analysis on DEGs involved in cellular component in leaf blades of sweet sorghum after 200 mM NaCl treatment for 48 h. The X axis indicates the percentage of GO termed genes among all DEGs, the Y axis indicated GO terms. The size of dots reflects the number of genes, the color of dots reflects the *q* value of hypergeometric test.

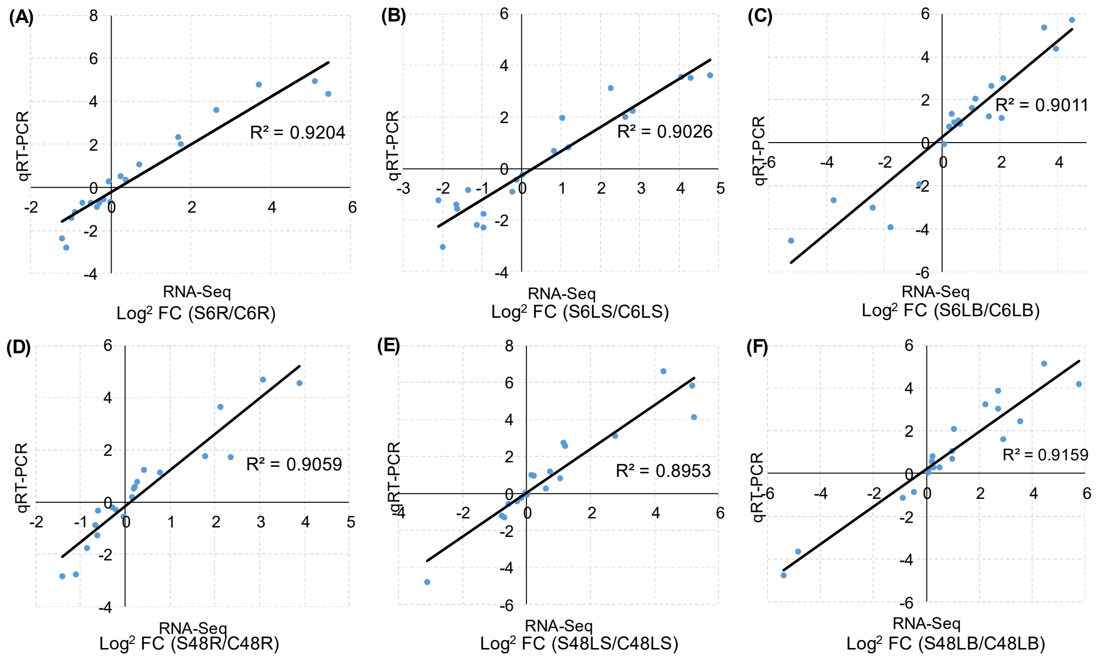

Figure S8 Correlation analysis for expression pattern validation of 20 randomly selected DEGs under 200 mM NaCl treatment for 6 h and 48 h in roots ((**A**, **D**), respectively), leaf sheaths ((**B**, **E**), respectively), and leaf blades ((**C**, **F**), respectively) by qRT-PCR method. The X-axes and Y-axes show the gene transcript level changes obtained by RNA-seq and qRT-PCR, respectively. R^2 indicates the correlation.
